# Outcomes following SARS-CoV-2 infection in liver transplant recipients: an international registry study

**DOI:** 10.1016/S2468-1253(20)30271-5

**Published:** 2020-08-28

**Authors:** Gwilym J Webb, Thomas Marjot, Jonathan A Cook, Costica Aloman, Matthew J Armstrong, Erica J Brenner, Maria-Andreea Catana, Tamsin Cargill, Renumathy Dhanasekaran, Ignacio García-Juárez, Hannes Hagström, James M Kennedy, Aileen Marshall, Steven Masson, Carolyn J Mercer, Ponni V Perumalswami, Isaac Ruiz, Sarang Thaker, Nneka N Ufere, Eleanor Barnes, Alfred S Barritt, Andrew M Moon

**Affiliations:** a Oxford Liver Unit, Translational Gastroenterology Unit, Oxford University Hospitals NHS Foundation Trust, University of Oxford, Oxford, UK; b Centre for Statistics in Medicine, University of Oxford, Oxford, UK; c Department of Medicine, Section of Hepatology, Rush University Medical Center, Chicago, IL, USA; d Liver Unit, Queen Elizabeth Hospital Birmingham, Birmingham, UK; e Division of Pediatric Gastroenterology and Hepatology, University of North Carolina, Chapel Hill, NC, USA; f Division of Gastroenterology and Hepatology, University of North Carolina, Chapel Hill, NC, USA; g Division of Gastroenterology/Hepatology, Department of Medicine, Beth Israel Deaconess Medical Center, Harvard Medical School, Boston, MA, USA; h Liver Center, Gastrointestinal Division, Massachusetts General Hospital, Harvard Medical School, Boston, MA, USA; i Department of Medicine, Stanford University School of Medicine, Palo Alto, CA, USA; j Department of Gastroenterology, Instituto Nacional de Ciencias Médicas y Nutrición Salvador Zubirán, Mexico City, Mexico; k Division of Hepatology, Department of Upper Gastrointestinal Diseases, Karolinska University Hospital, Stockholm, Sweden; l Sheila Sherlock Liver Unit, Royal Free Hospital, London, UK; m Liver Transplant Unit, Freeman Hospital, The Newcastle upon Tyne Hospitals NHS Foundation Trust, Newcastle upon Tyne, UK; n Translational and Clinical Research Institute, Newcastle University, Newcastle upon Tyne, UK; o Division of Liver Diseases, Department of Medicine, Icahn School of Medicine at Mount Sinai, New York, NY, USA; p Centre Hospitalier de l'Université de Montréal, Montréal, QC, Canada; q Division of Gastroenterology and Hepatology, University of Illinois at Chicago, Chicago, IL, USA

## Abstract

**Background:**

Despite concerns that patients with liver transplants might be at increased risk of adverse outcomes from COVID-19 because of coexisting comorbidities and use of immunosuppressants, the effect of severe acute respiratory syndrome coronavirus 2 (SARS-CoV-2) infection on this patient group remains unclear. We aimed to assess the clinical outcomes in these patients.

**Methods:**

In this multicentre cohort study, we collected data on patients with laboratory-confirmed SARS-CoV-2 infection, who were older than 18 years, who had previously received a liver transplant, and for whom data had been submitted by clinicians to one of two international registries (COVID-Hep and SECURE-Cirrhosis) at the end of the patient's disease course. Patients without a known hospitalisation status or mortality outcome were excluded. For comparison, data from a contemporaneous cohort of consecutive patients with SARS-CoV-2 infection who had not received a liver transplant were collected from the electronic patient records of the Oxford University Hospitals National Health Service Foundation Trust. We compared the cohorts with regard to several outcomes (including death, hospitalisation, intensive care unit [ICU] admission, requirement for intensive care, and need for invasive ventilation). A propensity score-matched analysis was done to test for an association between liver transplant and death.

**Findings:**

Between March 25 and June 26, 2020, data were collected for 151 adult liver transplant recipients from 18 countries (median age 60 years [IQR 47–66], 102 [68%] men, 49 [32%] women) and 627 patients who had not undergone liver transplantation (median age 73 years [44–84], 329 [52%] men, 298 [48%] women). The groups did not differ with regard to the proportion of patients hospitalised (124 [82%] patients in the liver transplant cohort *vs* 474 [76%] in the comparison cohort, p=0·106), or who required intensive care (47 [31%] *vs* 185 [30%], p=0·837). However, ICU admission (43 [28%] *vs* 52 [8%], p<0·0001) and invasive ventilation (30 [20%] *vs* 32 [5%], p<0·0001) were more frequent in the liver transplant cohort. 28 (19%) patients in the liver transplant cohort died, compared with 167 (27%) in the comparison cohort (p=0·046). In the propensity score-matched analysis (adjusting for age, sex, creatinine concentration, obesity, hypertension, diabetes, and ethnicity), liver transplantation did not significantly increase the risk of death in patients with SARS-CoV-2 infection (absolute risk difference 1·4% [95% CI −7·7 to 10·4]). Multivariable logistic regression analysis showed that age (odds ratio 1·06 [95% CI 1·01 to 1·11] per 1 year increase), serum creatinine concentration (1·57 [1·05 to 2·36] per 1 mg/dL increase), and non-liver cancer (18·30 [1·96 to 170·75]) were associated with death among liver transplant recipients.

**Interpretation:**

Liver transplantation was not independently associated with death, whereas increased age and presence of comorbidities were. Factors other than transplantation should be preferentially considered in relation to physical distancing and provision of medical care for patients with liver transplants during the COVID-19 pandemic.

**Funding:**

European Association for the Study of the Liver, US National Institutes of Health, UK National Institute for Health Research.

## Introduction

The COVID-19 pandemic has created a tension between the provision of long-term care for patients with chronic illness and the protection of those same patients from exposure to the causative virus, severe acute respiratory syndrome coronavirus 2 (SARS-CoV-2). Liver transplant recipients exemplify this tension, with a high prevalence of comorbidity combined with uncertainty regarding the effects of immunosuppression on SARS-CoV-2.^1^, ^2^, ^3^ At present, the minimisation of physical clinical contact with liver transplant recipients has been recommended in guidelines, but few data are available about the specific risks and characteristics of SARS-CoV-2 infection in liver transplant recipients.^4^

To date, published studies on COVID-19 in liver transplant recipients have reached differing conclusions and interpretation has been limited by the inclusion of patients without laboratory confirmation of SARS-CoV-2 infection and by the absence of comparison to patients who have not undergone liver transplantation.^5^, ^6^, ^7^, ^8^, ^9^, ^10^ Generalisable data from international cohorts of liver transplant recipients are therefore urgently required to elucidate the effects of immunosuppressive regimens and transplant-associated comorbidities on outcomes. A greater understanding of such associations would allow better prognostication and guide the resumption of in-person clinical encounters for this patient group.

**Research in context**

**Evidence before this study**

Before the start of this study (March 26, 2020), we searched for relevant literature via PubMed. We imposed no search limitations on language or date of publication and included studies in both paediatric and adult populations. We used the search terms “liver transplant” AND “COVID-19”, OR “SARS-CoV-2”. This process yielded no relevant articles, highlighting the urgent need for large-scale international data characterising the effect of COVID-19 on liver transplant recipients. Since starting the project, a small number of single-centre case series and a European registry cohort have been published. However, these studies have not included a comparison population and have lacked the power to detect risk factors associated with poor outcomes.

**Added value of this study**

To our knowledge, this international study including submissions from 18 countries reports on the largest cohort of liver transplant recipients with laboratory-confirmed severe acute respiratory syndrome coronavirus 2 (SARS-CoV-2) infection to date. It is also the first study to offer direct comparisons with patients without liver transplants who have tested positive for SARS-CoV-2 within the same period. The findings show that liver transplant recipients are not at increased risk of death from COVID-19 when matched to patients without liver transplants with similar comorbidities. Risk factors associated with poor prognosis in patients with COVID-19 in the general population, such as age and renal function, appear to be more important in determining outcome than having undergone liver transplantation.

**Implications of all the available evidence**

Our findings should provide reassurance to patients, clinicians, and health policy makers that liver transplant does not confer major additional susceptibility to adverse outcomes from COVID-19. This should be considered when assessing the relative risks and benefits of delivering clinical follow-up and monitoring patients with liver transplants during the COVID-19 pandemic.

We report the results from two international reporting registries for liver transplant recipients with laboratory-proven SARS-CoV-2 infection, and offer direct comparison with a contemporaneous cohort of patients with SARS-CoV-2 infection and without liver transplants. To our knowledge, these registries represent the largest reported cohort of liver transplant recipients with SARS-CoV-2 infection to date. Our objectives were to characterise the presentation of SARS-CoV-2 infection in liver transplant recipients, to identify risk factors associated with mortality, and to assess the potential association between liver transplantation and death among individuals with SARS-CoV-2 infection.

## Methods

### Study design and setting

We did a multicentre cohort study using an open online reporting form. Data were collected through two collaborating online registries: the COVID-Hep registry, coordinated by the University of Oxford (Oxford, UK); and the SECURE-Cirrhosis registry, coordinated by University of North Carolina (Chapel Hill, NC, USA). The registries were widely advertised through the communication channels of endorsing gastroenterology and hepatology societies, direct emails to hepatology providers, and through social media (appendix p 3). Clinicians were instructed to submit clinical data through an online case report form at the end of their patient's disease course (defined as the resolution of clinical signs of COVID-19, discharge from hospital, or death). Reporting clinicians were encouraged to submit all qualifying cases from their institution, but this could not be guaranteed. A copy of the data collection tool is available in the appendix (pp 20–38) and was identical for both registries.

To provide a comparison cohort, data were extracted with use of an identical data collection tool from the electronic patient records of consecutive patients who tested positive for SARS-CoV-2 over the same time period at Oxford University Hospitals National Health Service (NHS) Foundation Trust, an organisation of four hospitals in and around Oxford, UK. Positive cases from these hospitals were defined by the detection of SARS-CoV-2 by RT-PCR analysis of nasopharyngeal swab samples following the patient's presentation to hospital (including the emergency department, day cases, inpatients, and ambulatory services), but did not include community testing.

To minimise potential reporting bias, data extraction for the non-liver transplant cohort was done by investigators masked to the clinical characteristics and outcomes reported in the liver transplant cohort. All data for the liver transplant and non-liver transplant cohorts were uploaded in real time to the same secure, online data capture tool.^11^

All submitted report forms were manually reviewed to assess for data quality, completeness, and inconsistencies. In some instances, submitting clinicians were contacted and asked to provide additional data where appropriate.

The data collected contained no personal health identifiers, and both registries were deemed not to constitute human research by the University of Oxford Clinical Trials and Research Governance or the University of North Carolina Office of Human Research Ethics. We received formal local audit approval for data acquisition from the electronic health records of the Oxford University Hospitals NHS Foundation Trust (reference OUH5595).

### Participants

All cases of laboratory-confirmed SARS-CoV-2 infection in patients who had undergone liver transplantation and were older than 18 years of age, from any location, and with any symptom profile or level of disease severity, were included in the liver transplant cohort. Patients were excluded if any of the following conditions were met: SARS-CoV-2 infection was not confirmed in the laboratory, patients had not previously undergone liver transplantation, the submission was a duplicate, hospitalisation status or mortality outcome was not known or not reported, or the patient was aged 18 years or younger at the time of diagnosis. The current study includes 39 liver transplant recipients who were included in a previously published preliminary analysis.^8^ For the non-liver transplant cohort, those under 18 years of age, those missing data on mortality or hospitalisation status, or those with duplicate records were excluded. Patients in the non-liver transplant cohort found to have undergone liver transplantation were included in the liver transplant cohort.

### Variables and definitions

For the assessment of biochemical evidence of liver injury, the definitions of mild, moderate, and severe liver injury described by Phipps and colleagues were used.^12^ Specifically, we defined mild liver injury as a peak serum alanine aminotransferase (ALT) activity greater than 40 IU/L, moderate liver injury as a peak ALT of greater than 80 IU/L, and severe liver injury as a peak ALT of greater than 200 IU/L, with reported baseline values below these ranges. Acute kidney injury was defined as stage 2 if there was a doubling or greater increase of serum creatinine from baseline, and stage 3 if there was a tripling or greater increase of serum creatinine from baseline or a new requirement for renal replacement therapy, consistent with RIFLE criteria.^13^ Severity definitions for liver and kidney injury were not mutually exclusive.

Obesity was defined as a body-mass index of greater than 30 kg/m^2^. Where data on body-mass index were unavailable and obesity was not mentioned in medical records, obesity was assumed to be absent (in eight [5%] of liver transplant cohort and 133 [21%] of the non-liver transplant cohort). For analysis of ethnicity, only white ethnicity (as the majority classification) as compared with all other ethnicities was considered in the analysis. For the non-liver transplant cohort, where ethnicity was not recorded, white ethnicity was assumed.^14^

Serum creatinine was converted to mg/dL for the analysis, and baseline values taken before the diagnosis of SARS-CoV-2 were used.

### Outcomes

The major outcome of interest was death. We also examined rates of hospitalisation, requirement for intensive care, intensive care unit (ICU) admission, and invasive ventilation, and collected data on symptoms at presentation. For analysis, the composite outcome of requirement for intensive care was defined as either being admitted to an ICU or requiring intensive care on the basis of disease severity but being considered inappropriate because of other patient factors.

### Statistical analysis

Patient factors and outcome are summarised for both cohorts by occurrence of mortality using standard summary statistics (n [%] for binary measures and median [IQR] for continuous measures). The rates of missing data for each variable are reported. Univariable analysis of mortality by patient characteristic and multivariable comparisons of factors associated with death within the cohorts were done with logistic regression. The Hosmer-Lemeshow goodness-of-fit test was done for the multivariable logistic regression models. Only patients with data available for each reported data point (with the exceptions of values assumed for obesity and ethnicity explained above) were used in multivariable analyses; covariables showing collinearity with outcomes were excluded. Newcombe's method 10 was used to generate 95% CIs of the difference in proportions, along with Fisher's exact test to crudely compare outcomes between those who had received a transplant and those who had not. Nominal statistical significance at two-sided 5% level was used for the comparison of outcomes.

To compare the effect of liver transplantation on risk of death, 1:1 propensity score-matched samples (using a nearest neighbour approach) were constructed, with death as the outcome and transplantation status as the treatment variable. Covariates included in the propensity-score model were those thought to be independently associated with mortality for patients with SARS-CoV-2 infection (ie, age [years], sex, ethnicity, obesity, diabetes, hypertension, and baseline serum creatinine concentration [mg/dL]). Only patients with data available for each reported data point were included. Varying combinations of variables, and tests for interactions between variables, were assessed as reported in an attempt to generate propensity-score distributions that did not generate variance ratios less than 0·5 or greater than 2·0, with adjustments to the models aiming for a variance ratio for each covariable that was closer to 1·0. Propensity-score matching was done using the teffects function in Stata. The average treatment effect on the treated was calculated with robust Abaide-Imbens standard errors.^15^

All statistical analyses were done using Stata version 15.1.

### Role of the funding source

The funder of the study had no role in study design, data collection, data analysis, data interpretation, or writing of the report. The corresponding author had full access to all the data in the study and had final responsibility for the decision to submit for publication.

## Results

**Figure 1:**
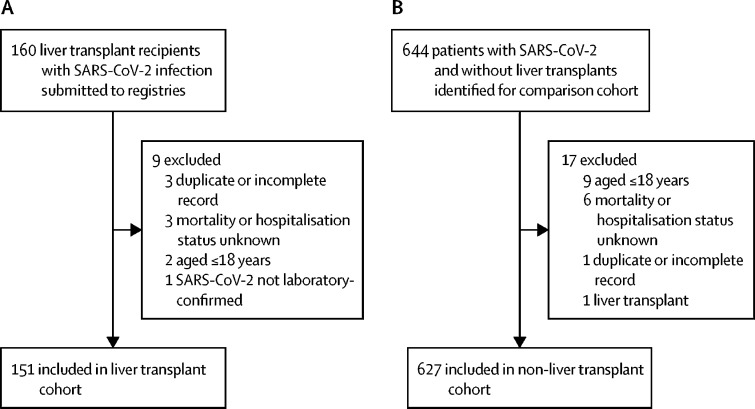
Cohort selection (A) Liver transplant cohort. (B) Comparison cohort. SARS-CoV-2= severe acute respiratory syndrome coronavirus 2.

Between March 25 and June 26, 2020, 160 reports of patients who were infected with SARS-CoV-2 infection and who had received liver transplants were submitted to the two registries. After exclusions, 151 liver transplant recipients remained (figure 1A). Within the same period, data were collected on 644 consecutive patients who had not received liver transplants and who tested positive for SARS-CoV-2 infection at the Oxford University Hospitals NHS Foundation Trust, of whom 627 remained after exclusions (figure 1B).

Of the 151 cases in the liver transplant cohort, the median age was 60 years (IQR 47–66), 102 (68%) patients were male and 49 (32%) were female, and 111 (74%) were of white ethnicity (table 1; appendix p 4). The cohort included patients from 18 countries, including the USA (55 [36%] patients), the UK (29 [19%]), Italy (15 [10%]), and one Middle Eastern country (18 [12%]; exact country withheld at contributor request; appendix p 5). 150 (99%) patients in the liver transplant cohort were taking immunosuppressants at the point of diagnosis of SARS-CoV-2 infection (table 1). Data on changes to immunosuppression regimens were not captured.

**Table 1:** Characteristics of the liver transplant and non-transplant comparison cohorts

			**Liver transplant cohort (n=151)**	**Comparison cohort (n=627)**	**p value**
Age, years			60 (47–66)	73 (55–84)	<0·0001
Sex			..	..	0·0010
	Male		102 (68%)	329 (52%)	..
	Female		49 (32%)	298 (48%)	..
Smoker			3 (2%)	7 (1%)	0·418
Ethnicity					
	White		111 (74%)	434 (69%)	0·324*
	Other ethnicities†		40 (26%)	193 (31%)	..
		African American	16 (11%)	NA	..
		Southeast Asian	11 (7%)	NA	..
		Hispanic	6 (4%)	NA	..
		East Asian	2 (1%)	NA	..
		Arab	2 (1%)	NA	..
		Unknown	4 (3%)	NA	..
Obesity‡			44 (29%)	158 (25%)	0·352
Cardiovascular disease			22 (15%)	202 (32%)	<0·0001
Diabetes			65 (43%)	144 (23%)	<0·0001
Asthma			0	69 (11%)	<0·0001
Chronic obstructive pulmonary disease			4 (3%)	59 (9%)	0·0043
Other chronic lung disease			4 (3%)	32 (5%)	0·279
Hypertension			63 (42%)	241 (38%)	0·459
Non-liver cancer			8 (5%)	92 (15%)	0·0011
Stroke or transient ischaemic attack			3 (2%)	73 (12%)	<0·0001
Serum creatinine concentration, mg/dL§			1·2 (0·9–1·5)	0·9 (0·7–1·1)	<0·0001
Immunosuppressant use			150 (99%)	19 (3%)	..
	Prednisolone or prednisone		67 (44%)	17 (3%)	<0·0001
	Calcineurin inhibitor		135 (89%)	6 (1%)	<0·0001
		Tacrolimus	127 (84%)	4 (1%)	<0·0001
		Ciclosporin	8 (5%)	2 (<1%)	<0·0001
	Antimetabolite		90 (60%)	7 (1%)	<0·0001
		Mycophenolate mofetil	77 (51%)	6 (1%)	<0·0001
		Azathioprine	13 (9%)	1 (<1%)	<0·0001
	Sirolimus		7 (5%)	0	<0·0001
SARS-CoV-2-targeted therapy			49 (32%)	17 (3%)	<0·0001
	Chloroquine or hydroxychloroquine		38 (25%)	5 (1%)	<0·0001
	Lopinavir or ritonavir		9 (6%)	5 (1%)	0·0003
	Remdesivir		6 (4%)	2 (<1%)	0·0010
	Oseltamivir		3 (2%)	0	0·0072
	Anakinra		2 (1%)	0	0·037
	Convalescent plasma		2 (1%)	0	0·037
	Tocilizumab		2 (1%)	0	0·037
	Azithromycin		1 (1%)	0	0·194
	Heparin¶		1 (1%)	0	0·194
	Sofosbuvir		1 (1%)	0	0·194
	Dexamethasone		0	1 (<1%)	1·000
	Interferon alfa		0	3 (<1%)	1·000
	Interferon beta		0	1 (<1%)	1·000
	Intravenous immunoglobulin		0	1 (<1%)	1·000

Data are median (IQR) or n (%). Comparisons with Mann-Whitney U tests or Fisher's exact test as appropriate. NA=not available. SARS-CoV-2= severe acute respiratory syndrome coronavirus 2.

* For white ethnicity versus other ethnicities grouped.

† One patient had two recorded ethnicities.

‡ Body-mass index >30 kg/m^2^.

§ Data were missing for 7 patients in the transplant group and 81 in the non-transplant group.

¶ Explicit use of heparin as therapy for SARS-CoV-2 infection.

The contributing aetiology of liver disease before transplantation included hepatitis C virus infection (26 [17%] patients), non-alcoholic steatohepatitis (20 [13%]), primary sclerosing cholangitis (19 [13%]), alcohol-associated liver disease (19 [13%]), and chronic hepatitis B virus infection (14 [9%]; appendix p 6). Four (3%) patients had received a second liver transplant. The median time from liver transplantation was 5 years (IQR 2–11). The most common comorbidities included diabetes, hypertension, obesity, and cardiovascular disease (table 1).

Within the liver transplant cohort, 49 (32%) patients were reported as having received specific therapy targeting SARS-CoV-2 (table 1). Because it was the only treatment used in a substantial number of patients (38 [25%]), only hydroxychloroquine was included in further analyses.

Of the 627 patients in the comparison cohort, the median age was 73 years (IQR 55–84), 329 (52%) patients were male and 298 (48%) were female (table 1). 19 (3%) patients who had not received liver transplants were taking immunosuppressants at the point of SARS-CoV-2 diagnosis (table 1). Three (<1%) patients had undergone renal transplantation and six (1%) had liver cirrhosis.

Significant differences were found between the liver transplant cohort and the comparison cohort: the liver transplant cohort was younger, had a higher proportion of men, had higher median baseline serum creatinine, and had a higher proportion of patients with diabetes (table 1). The liver transplant cohort also had lower proportions of patients with cardiovascular disease, asthma, chronic obstructive pulmonary disease, non-liver cancer, and stroke or transient ischaemic attack (table 1). As expected, use of each of the immunosuppressive drugs recorded was significantly greater in the liver transplant cohort (table 1).

Data on presenting symptoms were complete in 149 (99%) liver transplant recipients and 529 (84%) patients in the comparison cohort (appendix p 8). The proportion of patients with gastrointestinal symptoms (abdominal pain, diarrhoea, nausea, or vomiting) was higher among liver transplant recipients (45 [30%]) than among the comparison cohort (62 [12%]; p<0·0001). However, the presence of respiratory symptoms at diagnosis did not differ significantly between the liver transplant cohort (114 [77%]) and the comparison cohort (431 [81%]; p=0·248; appendix p 8). In propensity score-matched models, the frequency of gastrointestinal symptoms differed whereas rates of respiratory symptoms were similar between groups (appendix p 9).

**Figure 2:**
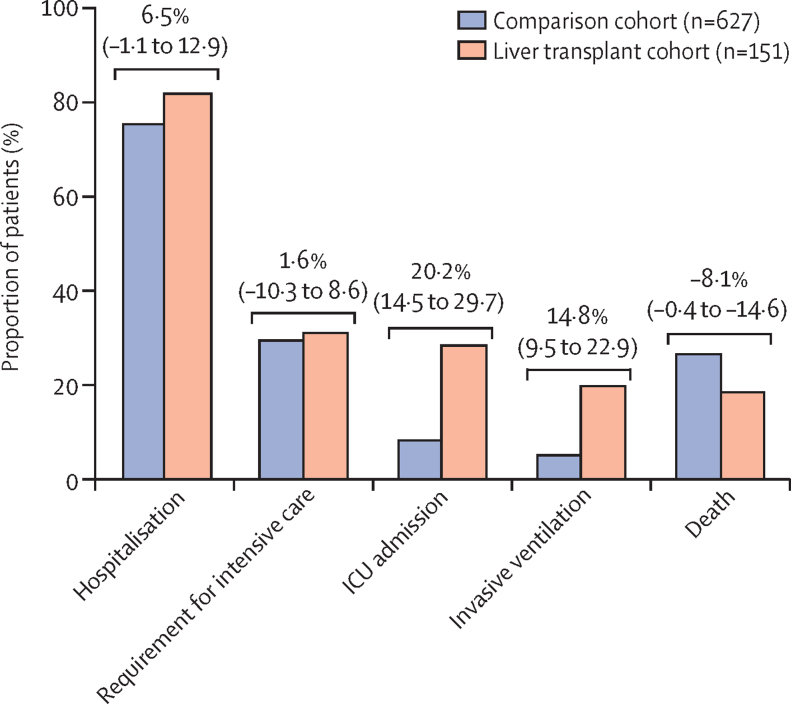
Major outcomes from severe acute respiratory syndrome coronavirus 2 infection in patients who have (n=151) and have not (n=627) undergone liver transplantation Risk differences between groups are presented with 95% CIs and were calculated with Newcombe's method 10. ICU=intensive care unit.

The proportion of patients hospitalised was similar between the liver transplant cohort (124 [82%]) and the comparison cohort (474 [76%]; p=0·106), but the proportions of patients admitted to the ICU (43 [28%] *vs* 52 [8%]; p<0·0001), and given invasive ventilation (30 [20%] *vs* 32 [5%]; p<0·0001) were higher in patients who had received liver transplants than in those who had not (figure 2).

Disease was deemed severe enough to warrant ICU admission but was otherwise considered inappropriate in three (2%) liver transplant recipients and 133 (21%) patients who had not received a liver transplant. In addition, one liver transplant recipient was recorded as being declined ICU admission because of resource availability, whereas no non-transplant patients were declined for this reason. When considering the total proportion of patients who were deemed to warrant ICU admission, whether admitted or not, the liver transplant cohort (47 [31%]) and the comparison cohort (185 [30%]) were similar (p=0·837; figure 2). For this composite outcome of requirement for ICU admission, multiple logistic regression analysis (considering age [years], sex, ethnicity, obesity, diabetes, hypertension, and baseline serum creatinine concentration [mg/dL] as covariables; data not shown) showed that only age was significantly associated (odds ratio [OR] 1·04 [95% CI 1·00–1·09] per 1 year increase, p=0·035).

After admission to the ICU, 20 (47%) of 43 patients in the liver transplant cohort and 18 (35%) of 52 in the comparison cohort died (p=0·294). Similarly, the proportion of patients who died after receiving invasive mechanical ventilation was non-significantly higher in the liver transplant cohort (16 [53%] of 30) than in the comparison cohort (12 [38%] of 32; p=0·307).

Overall, the proportion of patients who died in the liver transplant cohort (28 [19%]) was significantly lower than that in the comparison cohort (167 [27%]; p=0·046). The dominant cause of death in both groups was COVID-19 lung disease (21 [75%] of deaths in the liver transplant cohort, and 146 [89%] in the comparison cohort). Causes of death for each group are presented in the appendix (p 10). No deaths were liver-related in the transplant group. In the transplant cohort, six (75%) of the eight patients with non-liver cancer died, among whom the causes of death were COVID-19 lung disease (four [67%]), cardiac-related (one [17%]), and multiorgan failure (one [17%]).

For patients who were discharged, median duration of hospital stay was longer among liver transplant recipients (11 days [IQR 6–16]) than among those in the comparison cohort (8 days [3–13]; p=0·0046), with data available for 79 (81%) of 97 patients in the liver transplant cohort and 297 (94%) of 316 in the comparison cohort.

**Figure 3:**
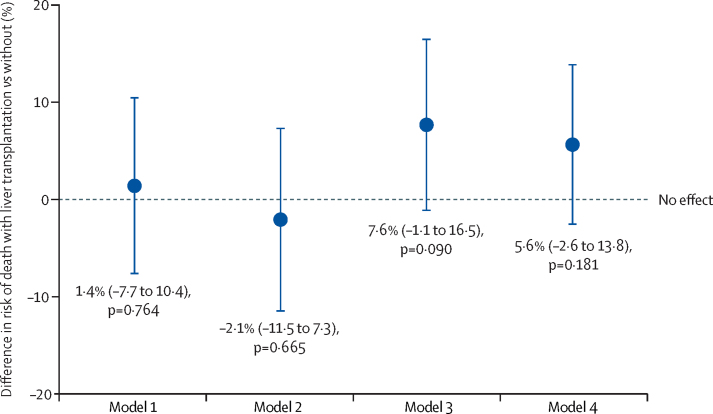
Propensity score-matched models for the association between liver transplantation and death in patients with severe acute respiratory syndrome coronavirus 2 infection The plot shows four separate propensity-score matched models with liver transplantation as the treatment variable and death as the outcome variable. Risk difference (95% CI) is presented for each model. For model 1, variables included in the calculation of propensity score were age, sex, obesity, white ethnicity, hypertension, diabetes, and serum creatinine. Subsequent models also included interactions with age (model 2), interactions with serum creatinine and age (model 3), and interactions with age but with serum creatinine concentration omitted (model 4). Seven (5%) of 151 transplant patients lacked baseline data for serum creatinine and were not included in models including serum creatinine. Further details are provided in the appendix (pp 9, 11).

To test for an association between liver transplantation and death, a propensity score-matched model was constructed, with transplantation status as the binary treatment variable and death as the binary outcome variable, and age (years), sex, ethnicity, obesity, diabetes, hypertension, and baseline serum creatinine concentration (mg/dL) included as covariates (model 1). Model 1 had variance ratios of 0·65 for age and 1·3 for creatinine, so a subsequent adjustment was made for interactions with age (model 2). This yielded a variance ratio of 1·08 for age but was more unbalanced for creatinine. Thus model 3 included interactions with both creatinine and age; however, matching for creatinine was poor. Model 4 was thus constructed including interaction with age but omitting creatinine (appendix p 11). Model 4 generated variance ratios within the 0·9–1·1 range for all variables. Notably, none of the analyses showed any independent association between liver transplantation and risk of death following SARS-CoV-2 infection (figure 3; appendix pp 16–19). Rates of other outcomes did not vary greatly from those seen in the total cohort (appendix pp 16–19).

**Figure 4:**
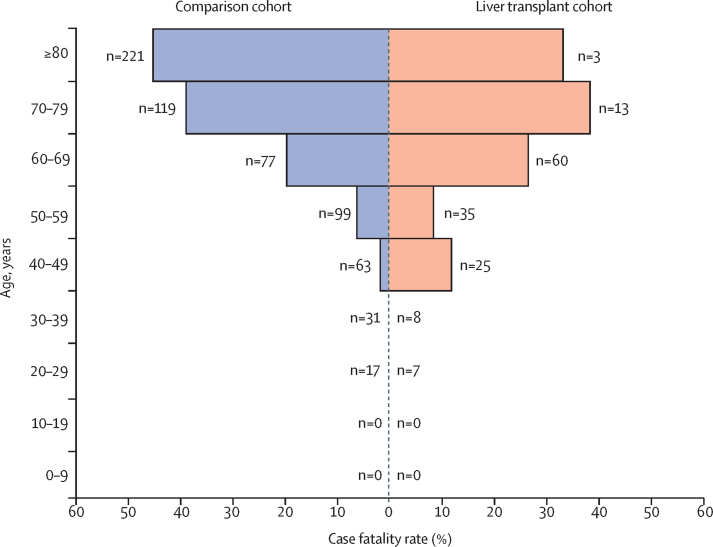
Case fatality rate of severe acute respiratory syndrome coronavirus 2 infection according to age group and liver transplantation status n denotes the total number of patients in each age group for each cohort.

Among liver transplant recipients, both univariable and multivariable analyses showed that the factors significantly associated with death were increased age (OR 1·06 [95% CI 1·01–1·11] per 1 year increase, p=0·031 on multivariable analysis), presence of non-liver cancer (18·30 [1·96–170·75]; p=0·011), and higher baseline serum creatinine (1·57 [1·05–2·36] per 1 mg/dL increase; p=0·028), whereas time from liver transplantation and immunosuppressant use showed no such association (table 2). Stepwise forwards or backwards selection of variables with a threshold of p<0·2 yielded similar findings (data not shown). Mortality varied by age band, with higher mortality in older patients, whether or not they had received a liver transplant (figure 4).

**Table 2:** Associations between patient characteristics and death in the liver transplant cohort

		Survived (n=123)*	Died (n=28)*	**Univariable analysis**		**Multivariable analysis**	
				OR (95% CI)	p value	OR (95% CI)	p value
Age (per 1-year increase)		57 (47–65)	66 (61–68)	1·07 (1·03–1·12)	<0·0001	1·06 (1·01–1·11)	0·031
Years from liver transplant (per 1 year increase)		4 (2–11)	9 (5–14)	1·05 (0·99–1·10)	0·086	1·04 (0·96–1·12)	0·393
Male sex (*vs* female)		83 (67%)	19 (68%)	1·02 (0·42–2·45)	0·969	0·79 (0·24–2·62)	0·702
Smoker (*vs* non-smoker)		2 (2%)	1 (4%)	2·24 (0·20–25·62)	0·516	0·95 (0·03–29·82)	0·978
White ethnicity (*vs* other ethnicities)		91 (74%)	20 (71%)	0·88 (0·35–2·19)	0·782	1·56 (0·45–5·42)	0·485
Obesity† (yes *vs* no)		34 (28%)	10 (36%)	1·45 (0·61–3·46)	0·398	1·10 (0·37–3·31)	0·864
Cardiovascular disease (yes *vs* no)		17 (14%)	5 (18%)	1·36 (0·45–4·05)	0·586	0·65 (0·15–2·71)	0·550
Diabetes (yes *vs* no)		51 (41%)	14 (50%)	1·41 (0·62–3·22)	0·412	1·83 (0·57–5·87)	0·310
Asthma (yes *vs* no)		0 (0%)	0 (0%)	..	..	..	..
Chronic obstructive pulmonary disease (yes *vs* no)		4 (3%)	0 (0%)	..	..	..	..
Other chronic lung disease (yes *vs* no)		1 (1%)	3 (11%)	1·36 (0·45–4·05)	0·586	13·04 (0·60–281·45)	0·101
Hypertension (yes *vs* no)		50 (41%)	13 (46%)	1·27 (0·55–2·89)	0·576	1·03 (0·34–3·09)	0·961
Non-liver cancer (yes *vs* no)		2 (2%)	6 (21%)	16·50 (3·13–87·09)	<0·0001	18·30 (1·96–170·75)	0·011
Stroke or transient ischaemic attack (yes *vs* no)		3 (2%)	0 (0%)	..	..	..	..
Serum creatinine concentration (per 1 mg/dL increase)		1·2 (0·9–1·4)	1·5 (1·1–2·0)	1·46 (1·07–1·97)	0·016	1·57 (1·05–2·36)	0·028
Prednisolone use (yes *vs* no)		56 (46%)	11 (39%)	0·77 (0·34–1·79)	0·549	1·74 (0·55–5·50)	0·345
Calcineurin inhibitor use (yes *vs* no)		109 (89%)	26 (93%)	1·67 (0·36–7·81)	0·515	3·73 (0·26–52·41)	0·329
	Tacrolimus use	105 (85%)	22 (79%)	..	..	..	..
	Ciclosporin use	4 (3%)	4 (14%)	..	..	..	..
Antimetabolite use (yes *vs* no)		75 (61%)	15 (54%)	0·74 (0·32–1·69)	0·472	0·68 (0·23–2·01)	0·484
	Mycophenolate mofetil use	64 (52%)	13 (46%)	..	..	..	..
	Azathioprine use	11 (9%)	2 (7%)	..	..	..	..
Sirolimus use (yes *vs* no)		7 (6%)	0 (0%)	..	..	..	..
Hydroxychloroquine treatment (yes *vs* no)		31 (25%)	7 (25%)	0·99 (0·38–2·55)	0·982	1·22 (0·34–4·36)	0·760

Associations between patient characteristics and death were assessed by univariable and multivariable logistic regression analyses. Multivariable analysis included all the variables for which results are presented in the table; other variables were excluded because of collinearity or absence of patients with the given characteristic in one group. Data were available (after assumptions detailed in the methods) for all patients in all categories, with the exception of missing baseline serum creatinine values in seven patients (excluded from multivariable analysis). The Hosmer-Lemeshow goodness-of-fit statistic was 0·684. OR=odds ratio.

* Data are median (IQR) or n (%).

† Body-mass index >30 kg/m^2^.

Within the non-liver transplant cohort, univariable analysis showed that increased age, male sex, white ethnicity, heart disease, diabetes, chronic obstructive pulmonary disease, other chronic lung disease, hypertension, and non-liver cancer were associated with death (appendix p 7). Age (OR 1·07 [95% CI 1·05–1·09] per 1 year increase**;** p<0·0001), male sex (1·89 [1·23–2·90]; p=0·0036), and diabetes (1·74 [1·08–2·80]; p=0·022) remained significant on multivariable analysis (appendix p 7).

Data on baseline and peak serum ALT activity were available for 121 (80%) patients in the liver transplant cohort and 448 (71%) in the comparison cohort (appendix p 12). The liver transplant and comparison cohorts did not differ with regard to the frequencies of mild liver injury (36 [30%] *vs* 126 [28%], p=0·734), moderate liver injury (19 [16%] *vs* 63 [14%], p=0·662), or severe liver injury (ten [8%] *vs* 17 [4%], p=0·052). Across both cohorts, ten (37%) of the 27 patients with severe liver injury and 150 (28%) of the 542 without severe liver injury died (p=0·281). Of those 27 with severe liver injury, six (22%) had received antiviral therapy.

Data on baseline and peak serum creatinine concentration were available for 129 (85%) patients in the liver transplant cohort and 537 (86%) of those in the comparison cohort. In addition, one patient in the liver transplant cohort who was missing baseline creatinine concentration data had a new requirement for renal replacement therapy (thus meeting the criteria for stage 3 acute kidney injury). There were no differences between cohorts in terms of the proportions of patients with stage 2 acute kidney injury (27 [21%] of 129 in the liver transplant cohort *vs* 94 [18%] of 537 in the comparison cohort, p=0·374) or stage 3 acute kidney injury (22 [17%] of 130 *vs* 74 [14%] of 537, p=0·403).

## Discussion

In this large, multinational cohort of liver transplant recipients with laboratory-confirmed SARS-CoV-2 infection, liver transplantation was not associated with an increased risk of mortality when compared with a contemporaneous cohort of patients who had not received a liver transplant. The proportions of liver transplant recipients who were hospitalised with SARS-CoV-2 infection (82%) and who died (19%) were similar to those in a previous European case series, which reported that 81% of patients were hospitalised and 16% died, with some patients in that series still part way through their disease course.^9^ Among liver transplant recipients in the current study, advanced age, increased baseline creatinine concentration, and the presence of non-liver cancer were independently associated with an increased risk of mortality. By contrast, the type of immunosuppressants used and the time from transplantation were not independently associated with mortality. In further contrast to findings from other, smaller cohorts, biological age rather than time from transplantation was most strongly correlated with death.^5^, ^6^, ^7^

Although the liver transplant cohort and the comparison cohort did not differ with regard to the proportions of patients who were hospitalised, who required ICU, or who died, ICU admission was more common in those with than in those without a liver transplant. These observations persisted in the propensity score-matched models. A potential explanation for this finding is that, compared with transplant recipients, a greater proportion of patients who had not received liver transplants were considered inappropriate for ICU admission, despite requiring it on the basis of disease severity, possibly because of concurrent comorbidity. Transplant recipients might also have been preferentially admitted to the ICU because of the previous time invested in their care and because the presence of life-limiting comorbidity would have been a contraindication to transplantation in the first instance.

In the propensity score-matched analysis, having received a liver transplant was not associated with death following SARS-CoV-2 infection. As such, although reasonable safeguards should remain in place to protect liver transplant recipients from SARS-CoV-2 infection, transplantation is not reason alone to delay other time-sensitive clinical care. Given that age and presence of comorbidities were significantly associated with risk of death in the cohorts of patients who had and had not undergone liver transplantation, greater emphasis should be placed on other coexisting comorbidities, rather than transplantation status per se, when risk-stratifying liver transplant recipients. Indirectly, these findings suggest that liver transplantation, where indicated, should not be delayed during the COVID-19 pandemic, and that supportive care should not be limited for patients with existing liver transplants with COVID-19.

We found that, upon diagnosis of SARS-CoV-2 infection, liver transplant recipients frequently had gastrointestinal symptoms, with 30% having abdominal pain, vomiting, or diarrhoea, compared with 12% of patients without liver transplant. Clinicians should therefore be vigilant and consider having a lower threshold for SARS-CoV-2 testing in transplant patients presenting with gastrointestinal symptoms. Frequencies of kidney injury and liver injury were not significantly different between patients who had and those who had not received a liver transplant, suggesting that transplanted livers were no more susceptible to injury and that calcineurin inhibitor-based immunosuppression was not associated with renal injury in SARS-CoV-2 infection.

Strengths of the current study include the international nature of liver transplant cases, low risk of misclassification of risk factors and outcomes because of clinician reporting, and comparisons with a large group of patients without liver transplants collected using the same methodology within the same timeframe. However, our findings must be interpreted in the context of this study's potential limitations. For instance, our transplant registry data were subject to reporting bias, potentially leading to over-representation of severe cases of COVID-19. However, our reported rates of hospitalisation and mortality are similar to those reported in previous studies, and a potential bias introduced towards the preferential submission of hospitalised patients to the registry serves to strengthen our conclusion that liver transplant recipients are not at increased risk of mortality compared with patients who had not received liver transplants. Additionally, in contrast to cases of liver transplant recipients being submitted internationally, the comparison cohort was limited to one region in the UK and therefore does not account for potential geographical differences in COVID-19 in patients without liver transplants. However, our comparison cohort does appear to be representative of those reported elsewhere, with risk factors associated with adverse outcomes similar to those widely reported in other large observational studies.^16^, ^17^, ^18^

A further limitation is that the overall sample, although substantial for the area, is not large enough rule out the possibility of a considerable difference in mortality (eg, up to 10%) between the groups, and because we did not collect time-to-event data we cannot comment on time-related outcomes. Furthermore, there were key differences in comorbidity between our comparison cohort and the liver transplant cohort, making direct comparisons challenging. It is also difficult to adjust fully for all comorbidities associated with liver transplantation, such as cardiovascular disease and renal insufficiency. We only collected data on a few laboratory variables, and had no data on stage of chronic kidney disease.

The relative predominance of white patients from Europe and North America limits the generalisability of these results to other populations, and further data from such populations are required. In addition, we had no data on how immunosuppression regimens were altered during the disease course of SARS-CoV-2 infection, if at all. It is possible that such changes could have affected the disease course, and are likely to have varied on a case-by-case basis.

We also cannot account for differences in approaches to diagnosis, likelihood of hospital admission, eligibility for high-dependence care, or overall quality of care between treating centres. This limitation also applies to our comparison cohort, and specifically to thresholds for ICU admission and declining ICU admission on the basis of comorbidity or other reasons. Antiviral therapies reported here might have been preferentially initiated in patients with more severe disease or in response to some other factor, and approaches are likely to have differed between submitting centres.

Lastly, although we attempted to collect data on major covariables, there remains a possibility of unmeasured confounding not captured by our report form. Relevant unmeasured confounders could include diet, physical activity, or fibrosis or cirrhosis in recipient grafts (although neither has been common in other series).^19^ The propensity-score analysis suggests some residual confounding, although the consistency of the findings across different models provided some reassurance. Despite these shortfalls, the current study describes the largest cohort of liver transplant recipients with SARS-CoV-2 infection to date, and overall it appears unlikely that transplantation has a major negative impact on outcome following SARS-CoV-2 infection.

In summary, this study involving more than 700 patients, including 151 liver transplant recipients, suggests that previous liver transplantation is not independently associated with death following SARS-CoV-2 infection. Conversely, advanced age and medical comorbidities such as renal impairment were associated with SARS-CoV-2-related mortality. Thus, traditional risk factors for adverse outcomes from COVID-19 should be preferentially considered when considering the risks and benefits of hospital attendance, immunosuppression, and social-distancing requirements for liver transplant recipients during the ongoing COVID-19 pandemic.

## References

[1] Watt KD, Pedersen RA, Kremers WK, Heimbach JK, Charlton MR (2010). Evolution of causes and risk factors for mortality post-liver transplant: results of the NIDDK long-term follow-up study. Am J Transplant.

[2] D'Antiga L (2020). Coronaviruses and immunosuppressed patients: the facts during the third epidemic. Liver Transpl.

[3] Wynants L, Van Calster B, Collins GS (2020). Prediction models for diagnosis and prognosis of covid-19 infection: systematic review and critical appraisal. BMJ.

[4] Boettler T, Newsome PN, Mondelli MU (2020). Care of patients with liver disease during the COVID-19 pandemic: EASL-ESCMID position paper. JHEP Rep.

[5] Bhoori S, Rossi RE, Citterio D, Mazzaferro V (2020). COVID-19 in long-term liver transplant patients: preliminary experience from an Italian transplant centre in Lombardy. Lancet Gastroenterol Hepatol.

[6] Pereira MR, Mohan S, Cohen DJ (2020). COVID-19 in solid organ transplant recipients: initial report from the US epicenter. Am J Transplant.

[7] Lee BT, Perumalswami PV, Im GY, Florman S, Schiano TD (2020). COVID-19 in liver transplant recipients: an initial experience from the U.S. epicenter. Gastroenterology.

[8] Webb GJ, Moon AM, Barnes E, Barritt AS, Marjot T (2020). Determining risk factors for mortality in liver transplant patients with COVID-19. Lancet Gastroenterol Hepatol.

[9] Belli LS, Duvoux C, Karam V (2020). COVID-19 in liver transplant recipients: preliminary data from the ELITA/ELTR registry. Lancet Gastroenterol Hepatol.

[10] Patrono D, Lupo F, Canta F (2020). Outcome of COVID-19 in liver transplant recipients: a preliminary report from northwestern Italy. Transpl Infect Dis.

[11] Harris PA, Taylor R, Minor BL (2019). The REDCap consortium: building an international community of software platform partners. J Biomed Inform.

[12] Phipps MM, Barraza LH, LaSota ED (2020). Acute liver injury in COVID-19: prevalence and association with clinical outcomes in a large US cohort. Hepatology.

[13] Hoste EA, Clermont G, Kersten A (2006). RIFLE criteria for acute kidney injury are associated with hospital mortality in critically ill patients: a cohort analysis. Crit Care.

[14] Collins GS, Altman DG (2010). An independent and external validation of QRISK2 cardiovascular disease risk score: a prospective open cohort study. BMJ.

[15] Garrido MM, Kelley AS, Paris J (2014). Methods for constructing and assessing propensity scores. Health Serv Res.

[16] Zhou F, Yu T, Du R (2020). Clinical course and risk factors for mortality of adult inpatients with COVID-19 in Wuhan, China: a retrospective cohort study. Lancet.

[17] Yang J, Zheng Y, Gou X (2020). Prevalence of comorbidities and its effects in patients infected with SARS-CoV-2: a systematic review and meta-analysis. Int J Infect Dis.

[18] Williamson E, Walker AJ, Bhaskaran KJ (2020). OpenSAFELY: factors associated with COVID-19-related hospital death in the linked electronic health records of 17 million adult NHS patients. medRxiv.

[19] Seyam M, Neuberger JM, Gunson BK, Hübscher SG (2007). Cirrhosis after orthotopic liver transplantation in the absence of primary disease recurrence. Liver Transpl.

