# Supplementary material for: Outcomes following SARS-CoV-2 infection in liver transplant recipients: an international registry study
Source: Lancet Gastroenterol Hepatol. 2020 Aug 28;5(11):1008–16. doi: 10.1016/S2468-1253(20)30271-5 (PMC7455160; doi:10.1016/S2468-1253(20)30271-5)
Supplement: Supplementary appendix [file mmc1.pdf]

THE LANCET
Gastroenterology & Hepatology

Supplementary appendix

This appendix formed part of the original submission and has been peer reviewed. We post it as supplied by the authors.

Supplement to: Webb GJ, Marjot T, Cook JA, et al. Outcomes following SARS-CoV-2 infection in liver transplant recipients: an international registry study. *Lancet Gastroenterol Hepatol* 2020; published online Aug 28. https://doi.org/10.1016/S2468-1253(20)30271-5.

Outcomes following SARS-CoV-2 infection in liver transplant recipients: an international registry study: Supplementary data

Supplementary tables

Supplementary table 1 – Supporting societies and routes of advertisement

**Society or route of advertisement**
European Association for the Study of the Liver
United European Gastroenterology
International Liver Cancer Association
British Society of Gastroenterology
British Association for the Study of the Liver
Hepatology Society of the Philippines
Gastroenterological Society of Australia
Australia and New Zealand Liver Transplant Registry
British Liver Trust
European Liver Patients' Association
Hellenic Association for the Study of the Liver
Twitter:
@COVIDHep
@SECUREcirrhosis

Supplementary table 2 – Reported ethnicity of transplant cases

Reported ethnicity	n	%
White	111	74%
African American	16	11%
South-East Asian	11	7%
Hispanic	6	4%
Unknown	4	3%
East Asian	2	1%
Arabic	2	1%

One patient had two recorded ethnicities

Supplementary table 3 – Countries of origin

Country	n	Percentage
USA	55	36%
UK	29	19%
Middle East*	18	12%
Italy	15	10%
Mexico	7	5%
Canada	6	4%
Sweden	5	3%
Belgium	3	2%
Netherlands	3	2%
Brazil	1	1%
Switzerland	1	1%
Germany	1	1%
Egypt	1	1%
Spain	1	1%
Greece	1	1%
India	1	1%
Philippines	1	1%
Portugal	1	1%
Turkey	1	1%

* = country known but unspecified at submitter's request.

Supplementary table 4 – Disease aetiologies requiring transplantation

Aetiologies	n	Percentage
HCV	26	17%
NASH	20	13%
Other	20	13%
Alcohol	19	13%
PSC	19	13%
HBV	14	9%
AIH	13	9%
PBC	6	4%
Alcohol HCV	2	1%
HBV Other	2	1%
Wilsons	2	1%
AIH PBC	1	1%
AIH Wilsons	1	1%
HCV HBV	1	1%
IGG4	1	1%
NASH Alcohol HCV	1	1%
NASH HBV	1	1%
NASH HCV	1	1%
NASH Other	1	1%

Supplementary table 5 – Characteristics and associations with death in comparison cohort

	Cohort (n = 627)		Survived (n = 469)		Died (n = 167)		Univariable analysis		Multivariable analysis	
	**Median/n**	**IQR/%**	**Median/n**	**IQR/%**	**Median/n**	**IQR/%**	**OR (95%CI)**	**p value**	**OR (95%CI)**	**p value**
**Age (years)**	73	73 (55-84)	63	63 (50-80)	82	82 (75-88)	1.07 (1.06-1.09)	**<0.001**	1.07 (1.05-1.09)	**<0.001**
**Sex (male)**	329	52.5%	227	49.3%	102	61.1%	1.61 (1.12-2.31)	**0.010**	1.89 (1.23-2.90)	**0.004**
**Smoker**	7	1.1%	6	1.3%	1	0.6%	0.46 (0.05-3.81)	0.469	0.38 (0.04-3.90)	0.415
**White ethnicity**	434	69.20%	296	64.40%	138	82.60%	2.64 (1.69-4.11)	**<0.001**	1.21 (0.71-2.06)	0.492
**Obese (BMI >30kg/m²)**	158	25.2%	121	26.3%	37	22.2%	0.80 (0.52-1.21)	0.291	1.06 (0.64-1.76)	0.810
**Heart disease**	202	32.2%	120	26.1%	82	49.1%	2.73 (1.89-3.95)	**<0.001**	1.03 (0.65-1.61)	0.910
**Diabetes mellitus**	144	23.0%	96	20.9%	48	28.7%	1.53 (1.02-2.29)	**0.039**	1.74 (1.08-2.80)	**0.022**
**Asthma**	69	11.0%	54	11.7%	15	9.0%	0.74 (0.41-1.35)	0.331	0.93 (0.46-1.89)	0.841
**Chronic obstructive pulmonary disease**	59	9.4%	35	7.6%	24	14.4%	2.04 (1.17-3.54)	**0.012**	1.10 (0.59-2.05)	0.771
**Other chronic lung disease**	32	5.1%	18	3.9%	14	8.4%	2.25 (1.09-4.63)	**0.028**	1.92 (0.84-4.41)	0.122
**Hypertension**	241	38.4%	164	35.7%	77	46.1%	1.54 (1.08-2.21)	**0.018**	1.01 (0.66-1.54)	0.978
**Non-liver cancer**	92	14.7%	56	12.2%	36	21.6%	1.98 (1.25-3.15)	**0.004**	1.55 (0.90-2.65)	0.112
**Stroke or TIA**	73	11.6%	48	10.4%	25	15.0%	1.51 (0.90-2.54)	0.119	0.90 (0.51-1.62)	0.736
**Chronic kidney disease**	95	15.2%	53	11.5%	42	25.1%	-	-	-	-
**Cirrhosis**	6	1.0%	0	0.0%	6	3.6%	-	-	-	-
**Other organ transplant**	3	0.5%	3	0.7%	0	0.0%	-	-	-	-
**Prednisolone use**	17	2.7%	11	2.4%	6	3.6%	1.52 (0.55-4.18)	0.416	1.19 (0.39-3.71)	0.758
**Tacrolimus use**	4	0.6%	4	0.9%	0	0.0%	-	-	-	-
**Ciclosporin use**	2	0.3%	2	0.4%	0	0.0%	-	-	-	-
**Sirolimus use***	0	0.0%	0	0.0%	0	0.0%	-	-	-	-
**Mycophenolate mofetil use**	6	1.0%	6	1.3%	0	0.0%	-	-	-	-
**Azathioprine use**	1	0.2%	1	0.2%	0	0.0%	-	-	-	-
**Serum creatinine (mg/dL)**	1	0.9 (0.7-1.1)	1	0.8 (0.7-1.1)	1	1.0 (0.8-1.3)	1.01 (0.87-1.16)	0.942	0.98 (0.79-1.21)	0.824

Hosmer-Lemeshow goodness of fit = 0.949; 546 of 627 patients included in multivariable analysis

Supplementary table 6 – Symptoms at diagnosis among transplant and non-transplant cohorts

Symptom profile	Non-transplant (529 of 627 with data)	Transplant (149 of 151 with data)	p value
**GI symptoms only**	12 (2%)	14 (9%)	-
**Respiratory symptoms only**	378 (71%)	83 (56%)	-
**Both respiratory & GI**	50 (9%)	31 (21%)	-
**Neither respiratory & GI**	89 (17%)	21 (14%)	-
**GI symptoms**	62 (12%)	45 (30%)	<0.001
**Respiratory symptoms**	431 (81%)	114 (77%)	0.248

Supplementary table 7 – Characteristics of groups after propensity score matching

	Model 1 (n=144)		Model 2 (n=144)		Model 3 (n=144)		Model 4 (n=151)	
	**Transplant**	**Non-transplant**	**Transplant**	**Non-transplant**	**Transplant**	**Non-transplant**	**Transplant**	**Non-transplant**
**Age (years)**	60 (47–66)	56 (46–66)	60 (47–66)	55 (48–64)	60 (47–66)	56 (47–66)	60 (47–66)	58 (47–65)
**Years after transplant**	5 (2–11)	-	5 (2–11)	-	5 (2–11)	-	5 (2–11)	-
**Sex (male)**	95 (66%)	94 (65%)	95 (66%)	94 (65%)	95 (66%)	101 (70%)	102 (67%)	108 (72%)
**Smoker**	3 (2%)	7 (5%)	3 (2%)	2 (1%)	3 (2%)	3 (2%)	3 (2%)	2 (1%)
**Ethnicity (white)**	105 (73%)	102 (71%)	105 (73%)	103 (72%)	105 (73%)	9 (67%)	111 (74%)	110 (73%)
**Heart disease**	21 (15%)	34 (24%)	21 (15%)	28 (19%)	21 (15%)	37 (26%)	22 (15%)	26 (17%)
**Diabetes mellitus**	61 (42%)	62 (43%)	61 (42%)	55 (38%)	61 (42%)	60 (42%)	65 (43%)	59 (39%)
**Hypertension**	57 (40%)	56 (39%)	57 (40%)	58 (40%)	57 (40%)	55 (28%)	63 (42%)	67 (44%)
**Cancer**	8 (5%)	13 (9%)	8 (5%)	13 (9%)	8 (5%)	12 (8%)	8 (5%)	5 (3%)
**Prednisolone**	64 (44%)	4 (3%)	64 (44%)	10 (7%)	64 (44%)	12 (8%)	67 (44%)	2 (1%)
**Calcineurin inhibitor use**	129 (90%)	2 (1%)	129 (90%)	10 (7%)	129 (90%)	18 (13%)	135 (89%)	2 (1%)
**Antimetabolite**	86 (60%)	1 (1%)	86 (60%)	8 (6%)	86 (60%)	14 (10%)	90 (60%)	2 (1%)
**Creatinine (mg/dl)**	1.2 (0.9–1.5)	0.9 (0.8–1.1)	1.2 (0.9–1.5)	0.9 (0.8–1.1)	1.2 (0.9–1.5)	1.0 (0.8–2.3	1.2 (0.9–1.5)	0.9 (0.8–1.1)
**Respiratory symptoms**	109 of 143 (76%)	101 of 121 (83%)	109 of 143 (76%)	117 of 133 (88%)	109 of 143 (76%)	92 of 127 (72%)	114 of 149 (77%)	100 of 120 (83%)
**Gastrointestinal symptoms**	45 of 143 (31%)	13 of 121 (11%)	45 of 143 (31%)	14 of 133 (11%)	45 of 143 (31%)	22 of 127 (17%)	30 of 149 (30%)	13 of 120 (11%)

Table shows four separate propensity-score matched models with liver transplantation as the treatment variable, death as the outcome variable. For Model 1, variables included for calculation of propensity score were age, sex, obesity, white ethnicity, hypertension, diabetes mellitus, and serum creatinine; for Model 2, interactions with age were added; for Model 3, interactions with serum creatinine were added; for Model 4, serum creatinine was removed. Seven of 151 transplant patients lacked baseline data for serum creatinine and were not included in models including serum creatinine.

Supplementary table 8 – Causes of death among transplant and non-transplant cohorts

Mode of death	Transplant cohort (n=28)	Non-transplant (n=164)
Respiratory	21 (75%)	146 (89%)
Hepatic	0 (0%)	0 (0%)
Cardiogenic	2 (7%)	3 (2%)
Other†	5 (18%)	15 (9%)

† = For LT patients, all ‘other’ causes of death represented multi-organ failure; among the non-transplant cohort, all those in the ‘other’ classification died from causes not immediately attributable to SARS-CoV-2 infection / COVID-19.

Supplementary table 9 – Standardised differences and variance ratio for unmatched and propensity score matched groups

	Model 1				Model 2				Model 3				Model 4			
Variables	UV	MV	USD	MSD	UV	MV	USD	MSD	UV	MV	USD	MSD	UV	MV	USD	MSD
**Age**	0.55	0.65	-0.9	0.09	0.55	1.27	-0.93	0.02	0.55	0.81	-0.93	0.07	0.49	1.03	-0.75	0.02
**Age#Age**	-	-	-	-	0.36	1.08	-1.02	0.05	0.36	0.79	-1.02	0.05	0.33	0.95	-0.86	0.02
**Sex**	0.91	0.99	0.3	0.01	0.91	0.99	0.26	0.01	0.91	1.07	0.26	-0.09	0.88	1.08	0.31	-0.09
**Obesity**	0.98	0.96	0	0.05	0.98	0.97	0.02	0.03	0.98	0.90	0.02	0.12	0.92	0.98	0.09	0.01
**White ethnicity**	1.11	0.94	0.1	0.07	1.11	0.93	0.10	-0.09	1.11	0.97	0.10	-0.03	1.10	0.99	0.09	-0.01
**Hypertension**	1.32	1.00	0.4	0.01	1.32	1.03	0.38	0.08	1.32	1.00	0.38	0.01	1.39	1.03	0.44	0.08
**Diabetes mellitus**	0.99	1.01	0	0.01	0.99	0.99	-0.03	-0.01	0.99	1.01	-0.03	0.03	1.03	0.99	0.07	-0.05
**Creatinine**	1.02	1.31	0.3	0.33	1.02	0.62	0.28	0.14	1.02	1.13	0.28	-0.04	-	-	-	-
**Creatinine#Creatinine**	-	-	-	-	-	-	-	-	0.46	2.94	0.07	0.00	-	-	-	-

Table shows four separate propensity-score matched models with liver transplantation as the treatment variable, death as the outcome variable. For Model 1, variables included for calculation of propensity score were age, sex, obesity, white ethnicity, hypertension, diabetes mellitus, and serum creatinine; for Model 2, interactions with age were added; for Model 3, interactions with serum creatinine were added; for Model 4, serum creatinine was removed. Seven of 151 transplant patients lacked baseline data for serum creatinine and were not included in models including serum creatinine. UV = unmatched variance, MV = matched variance, USD = unmatched standardised difference, MSD = matched standardised difference; - denotes omitted variable omitted. # denotes interactions.

Supplementary table 10 – Liver and renal biochemistry

Category	Transplant patients	Non-transplant patients	p value
**Mild liver injury** (ALT >ULN)	36 of 121 (30%)	126 of 448 (28%)	0.734
**Moderate liver injury** (ALT >2×ULN)	19 of 121 (16%)	63 of 448 (14%)	0.662
**Severe liver injury** (ALT >5×ULN)	10 of 121 (8%)	17 of 448 (4%)	0.052
**Renal injury stage 2** (>2 fold rise in creatinine from baseline)	27 of 129 (21%)	94 of 537 (18%)	0.374
**Renal injury stage 3** (>3 fold rise in creatinine from baseline or renal replacement therapy)	22 of 130 (17%)	74 of 537 (14%)	0.403

Supplementary table 11– Contributors

Contributor(s) and affiliation
Ahmed Hashim / David Patch, Royal Free Hospital, UK
Ahmed Hashim / Rachel Westbrook, Royal Free Hospital, UK
Ahmed Hashim / Aileen Marshall, Royal Free Hospital, UK
Ahmed Hashim / Jennifer Ryan, Royal Free Hospital, UK
Ahmed Tawheed / Mohamed El Kassas, Endemic Medicine Department Helwan University, Egypt
Andrea De Gottardi, Ente Ospedaliero Cantonale, Switzerland
Antonella Putignano / Thierry Gustot, CUB Erasme, Belgium
Beth Lusina / Kelly Burak, Foothills Medical Centre, Calgary, Alberta, Canada
Charmaine Matthews / Paul Richardson, Liverpool University Hospitals NHS Foundation Trust, UK
Costica Aloman / Nikunj Shah, Rush University Medical Center, USA
Costica Aloman / Sheila Eswaran, Rush University Medical Center, USA
Costica Aloman / Costica Aloman, Rush University Medical Center, USA
Daniele Nicolini / Marco Vivarelli, Division of Hepatobiliary, Pancreatic and Abdominal Transplant Surgery, A.O.U. "Ospedali Riuniti di Ancona", Italy
Daniele Nicolini / Marco Vivarelli, A.O.U. Ospedali Riuniti di Ancona, Ancona, Italy, Italy
Debbie Shawcross / Michael Heneghan, King's College Hospital, UK
Heather Lafferty / Kenneth Simpson, Scottish Liver transplant Unit, Edinburgh Royal Infirmary, UK
Matthew Armstrong / Ye Oo, Queen Elizabeth Birmingham / University Hospitals Birmingham, UK
Matthew Armstrong / James Ferguson, Queen Elizabeth Birmingham / University Hospitals Birmingham, UK
Roberta Elisa Rossi / Vincenzo Mazzaferro, Istituto Nazioanle dei Tumori, Milan, Italy
Sherrie Bhoori / Vincenzo Mazzaferro, Istituto Nazioanle dei Tumori, Milan, Italy
Holger Hinrichsen / Rainer Guenther, Department of Internal Medicine/Liver Unit, Germany
Elton Dajti / Mariarosa Tamè, Sant'Orsola Hospital, Italy
Felipe Alconchel / Pablo Ramírez, Virgen de la Arrixaca University Hospital, Spain
Feng Su / Iris Liou, University of Washington, USA
Graham Brady / Robert Fontana, University of Michigan, USA
Gupse Adali, SBU Umraniye Training and Research Hospital, Turkey
Gwilym Webb / Jane Collier, Oxford, UK
Hannes Hagström / Staffan Wahlin, Karolinska univ. sjukhus, Huddinge, Sweden
Hannes Hagström / Antti Oksanen, Karolinska University Hospital, Sweden
Hannes Hagström / Lina Lindström, Karolinska University Hospital, Sweden
Hannes Hagström, Karolinska University Hospital, Sweden
Ignacio García-Juárez, Instituto Nacional de Ciencias Médicas Nutrición Salvador Zubirán, Mexico
Isaac Ruiz / Hélène Castel, Centre Hospitalier de l'Université de Montréal (CHUM), Canada
Isaac Ruiz / Catherine Vincent, Centre Hospitalier de l'Université de Montréal (CHUM), Canada
Isaac Ruiz / Marc Bilodeau, Centre Hospitalier de l'Université de Montréal (CHUM), Canada
Jihane Benhamou, University of California Los Angeles, USA
Joost PH Drenth, UMCG Groningen, The Netherlands

Joost PH Drenth / Eric Tjwa, Erasmus MC Rotterdam, Netherlands, The Netherlands
Justin Boike / Josh Levitsky, Northwestern, USA
Justin Boike / Christopher Moore, Northwestern, USA
Justin Boike / Daniel Ganger, Northwestern, USA
Justin Boike, Northwestern, USA
Justin Chin / Varuna Aluvihare, King's College Hospital, UK
Justin Chin, King's College Hospital, UK
Katherine Marx / Maria Andreea Catana, Beth Israel Deaconess Medical Center, USA
Konstantina Nikitopoulou / Raaj Praseedom, Cambridge University Hospitals NHS Foundation Trust, UK
Konstantina Nikitopoulou / Paul Gibbs, Cambridge University Hospitals NHS Foundation Trust, UK
Lance L Stein / Lance L Stein, Piedmont Atlanta Hospital, USA
Logan Hobbs / Marwan Ghabril, IU Health University Health, USA
Maíra Godoy / Maíra Godoy, Santa Isabel Hospital, Brazil
Maria Andreea Catana / Micheal Curry, Beth Israel Deaconess Medical Center, USA
Mariarosa Tamè / Mariarosa Tamè, Sant'Orsola Hospital, Bologna, Italy
Matthew Armstrong / David Mutimer, Queen Elizabeth Birmingham / University Hospitals Birmingham, UK
Matthew Armstrong / Dhiraj Tripathi, Queen Elizabeth Birmingham / University Hospitals Birmingham, UK
Matthew Armstrong / Thamara, Queen Elizabeth Birmingham / University Hospitals Birmingham, UK
Michael Andrew Yu / Ram Subramanian, Emory Transplant Center, USA
Milena Mendes / Milena Mendes, Unidade de Transplante - Hospital Curry Cabral, Portugal
Monica Cucco / Gianluca Svegliati Baroni, Department of Gastroenterology, Polytechnic University of Marche, Ancona, Italy
Nikolas K. Gatselis / George N Dalekos, Department of Medicine and Research Laboratory of Internal Medicine, National Expertise Center of Greece in Autoimmune Liver Diseases, University Hospital of Larissa, Greece
Nneka Ufere / Shoko Kimura, Massachusetts General Hospital, USA
Nurun Tania / George Abouda, Hull University Teaching Hospitals, UK
Patricia D Jones / Martin, University of Miami-JMH, USA
Patricia D Jones / O'Brien, University of Miami-JMH, USA
Patricia D Jones, University of Miami-JMH, USA
Patricia D Jones, University of Miami-Jackson, USA
Ponni Perumalswami / Joseph Odin, Mount Sinai Hospital, USA
Ponni Perumalswami / Charissa Chang, Mount Sinai Hospital, USA
Ponni Perumalswami, Mount Sinai Hospital, USA
Ponni Perumalswami / Priya Grewal, Mount Sinai Hospital, USA
Ponni Perumalswami / Thomas Schiano, Mount Sinai Hospital, USA
Ponni Perumalswami / Jennifer Leong, Mount Sinai Hospital, USA
Ponni Perumalswami / Jawad Ahmad, Mount Sinai Hospital, USA
Ponni Perumalswami / Gene Im, Mount Sinai Hospital, USA
Ponni Perumalswami / Leona Kim-Schluger, Mount Sinai Hospital, USA
Ponni Perumalswami, New York University, USA
Rahul Maheshwari, Piedmont Atlanta Hospital, USA
Raymond Rubin / Rahul Maheshwari, Piedmont Atlanta Hospital, USA

S K Yadav / A S Soin, Medanta, The Medicity, India
Sarah Townsend / Douglas Thorburn, Royal Free Hospital, UK
Sarang Thaker / Adam Mikolajczyk, University of Illinois Hospital, USA
Sarang Thaker / Sean Koppe, University of Illinois Hospital, USA
Steven Masson / Mark Hudson, Newcastle, UK
Steven Masson, Newcastle, UK
Thines Karunakaran / Chirag Oza, Broomfield Hospital, UK
Vanessa de Villa, The Medical City, Philippines
Wim Laleman, University Hospitals Leuven, Belgium
Xavier Verhelst / Xavier Verhelst, Ghent University Hospital, Belgium
Zurabi Lominadze, University of Maryland Medical Center, USA

Supplementary figure 1 – Key outcomes after propensity score matching (model 1)

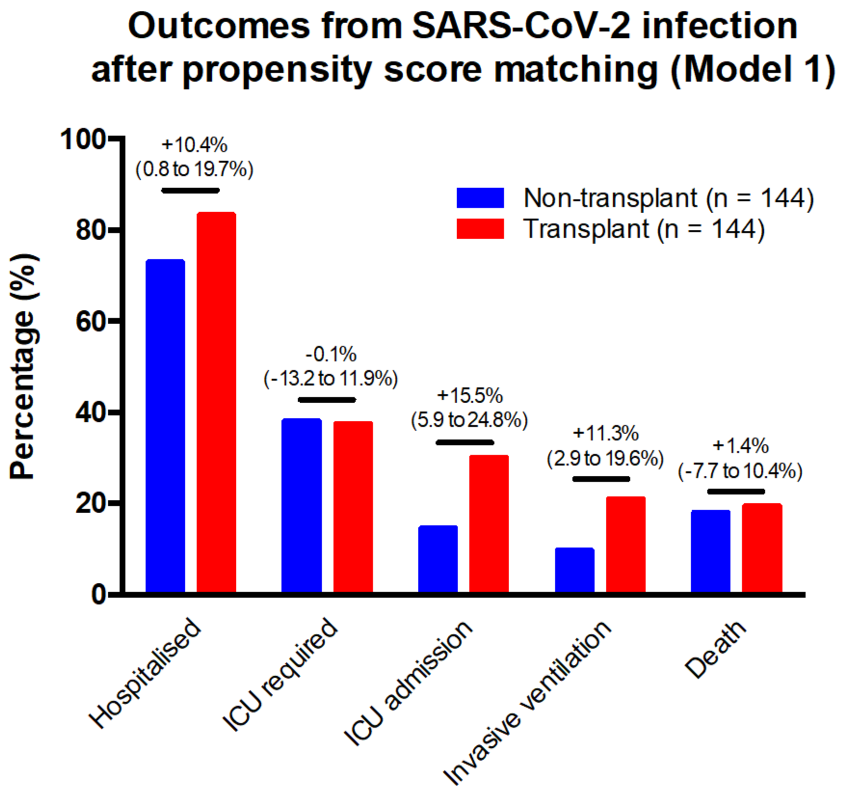

Comparison of major outcomes from SARS-CoV-2 infection between 144 liver transplant recipients and 144 patients without liver transplants after propensity score matching (model 1) based on age, sex, ethnicity, obesity, diabetes, hypertension and baseline serum creatinine. 95% confidence intervals for the difference in risk are shown in brackets and are calculated with Newcombe's method 10. ICU, intensive care unit.

Supplementary figure 2 – Key outcomes after propensity score matching (model 2)

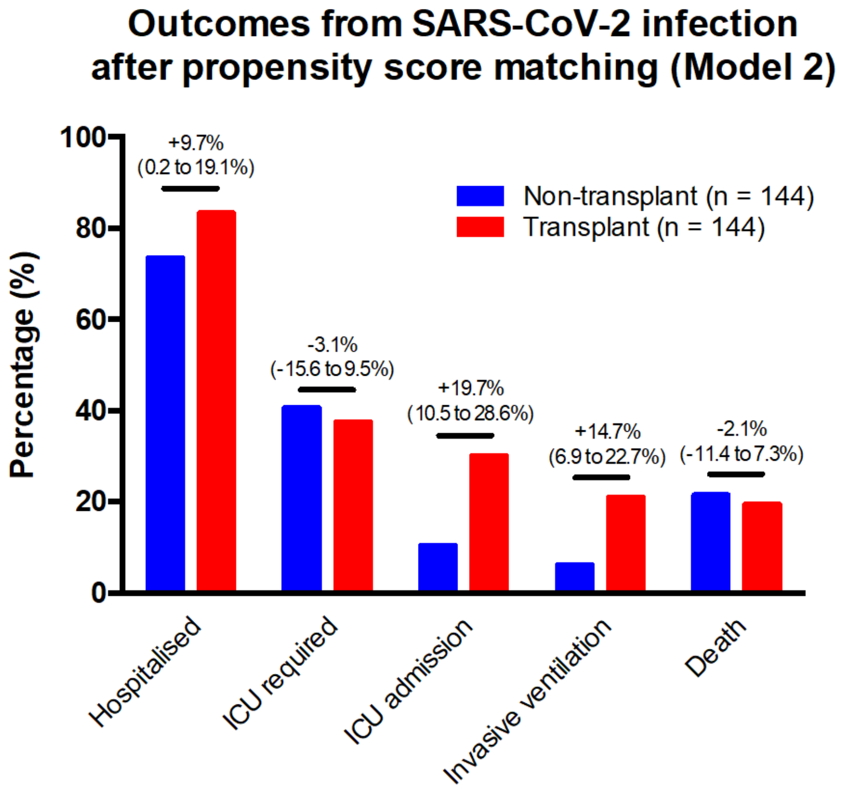

Comparison of major outcomes from SARS-CoV-2 infection between 144 liver transplant recipients and 144 patients without liver transplants after propensity score matching (model 2) based on age, interactions with age, sex, ethnicity, obesity, diabetes, hypertension and baseline serum creatinine. 95% confidence intervals for the difference in risk are shown in brackets and are calculated with Newcombe's method 10. ICU, intensive care unit.

Supplementary figure 3 – Key outcomes after propensity score matching (model 3)

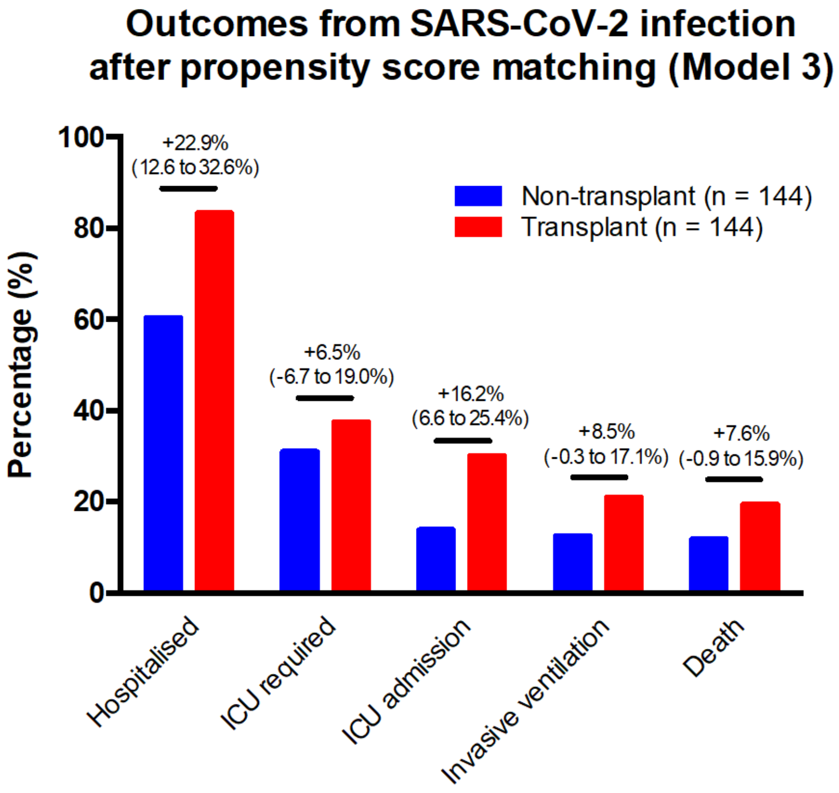

Comparison of major outcomes from SARS-CoV-2 infection between 144 liver transplant recipients and 144 patients without liver transplants after propensity score matching (model 3) based on age, interactions with age, sex, ethnicity, obesity, diabetes, hypertension, baseline serum creatinine, and interactions with baseline serum creatinine. 95% confidence intervals for the difference in risk are shown in brackets and are calculated with Newcombe's method 10. ICU, intensive care unit.

Supplementary figure 4 – Key outcomes after propensity score matching (model 4)

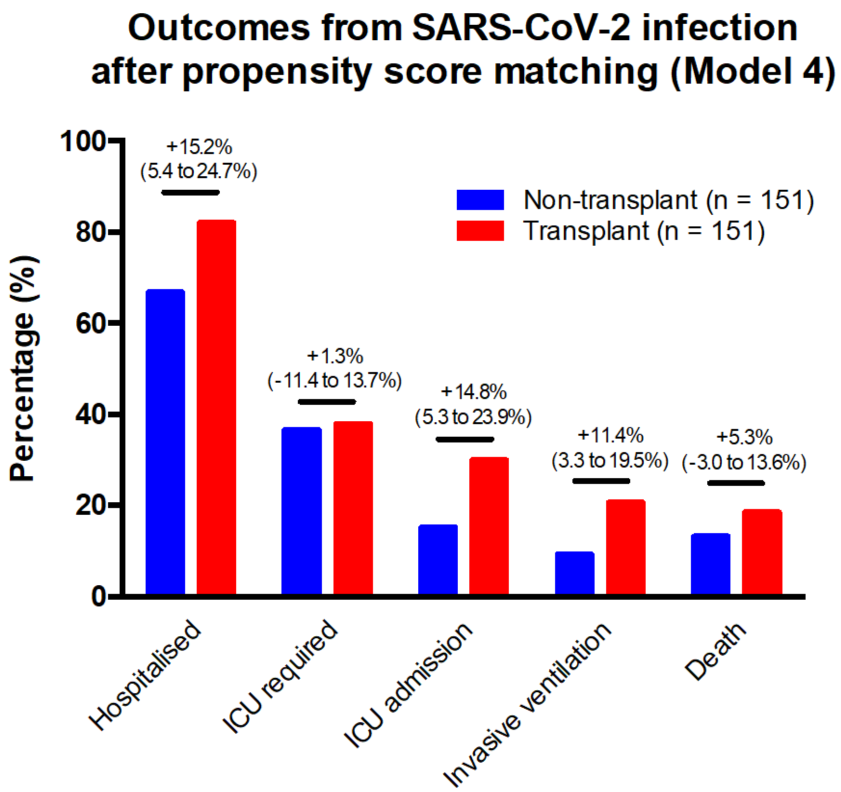

Comparison of major outcomes from SARS-CoV-2 infection between 151 liver transplant recipients and 151 patients without liver transplants after propensity score matching (model 4) based on age, interactions with age, sex, ethnicity, obesity, diabetes, and hypertension. 95% confidence intervals for the difference in risk are shown in brackets and are calculated with Newcombe's method 10. ICU, intensive care unit.

Supplementary appendix – Data collection tool

Inclusion Criteria:

1) Chronic liver disease or post-liver transplantation AND

2) Laboratory confirmed COVID 19 infection

Ideally this form should be completed after the patient has had COVID 19 for a long enough duration to experience complete recovery, discharge, or death.

If you have any questions, please reach out to info@covid-hep.net

Reporter Information

Name of reporter ______________________

Email address of reporter ______________________

Name of lead physician providing care for liver disease/post-liver transplant ______________________

Name of center providing care for liver disease/post-liver transplant ______________________

Name of hospital where patient received care for COVID 19 (enter 'NA' if patient not hospitalized) ______________________

Patient Information

Is the patient >90 years of age?
- ◯ Yes
- ◯ No

Age

- ◯ 89
- ◯ 88
- ◯ 87
- ◯ 86
- ◯ 85
- ◯ 84
- ◯ 83
- ◯ 82
- ◯ 81
- ◯ 80
- ◯ 79
- ◯ 78
- ◯ 77
- ◯ 76
- ◯ 75
- ◯ 74
- ◯ 73
- ◯ 72
- ◯ 71
- ◯ 70
- ◯ 69
- ◯ 68
- ◯ 67
- ◯ 66
- ◯ 65
- ◯ 64
- ◯ 63
- ◯ 62
- ◯ 61
- ◯ 60
- ◯ 59
- ◯ 58
- ◯ 57
- ◯ 56
- ◯ 55
- ◯ 54
- ◯ 53
- ◯ 52
- ◯ 51
- ◯ 50
- ◯ 49
- ◯ 48
- ◯ 47
- ◯ 46
- ◯ 45
- ◯ 44
- ◯ 43
- ◯ 42
- ◯ 41
- ◯ 40
- ◯ 39
- ◯ 38
- ◯ 37
- ◯ 36
- ◯ 35
- ◯ 34
- ◯ 33
- ◯ 32
- ◯ 31
- ◯ 30
- ◯ 29
- ◯ 28
- ◯ 27
- ◯ 26
- ◯ 25
- ◯ 24
- ◯ 23
- ◯ 22
- ◯ 21

◯ 20
◯ 19
◯ 18
◯ 17
◯ 16
◯ 15
◯ 14
◯ 13
◯ 12
◯ 11
◯ 10
◯ 9
◯ 8
◯ 7
◯ 6
◯ 5
◯ 4
◯ 3
◯ 2
◯ 1

Country of residence

◯ --- Not in this country list ---
◯ land Islands
◯ Afghanistan
◯ Albania
◯ Algeria
◯ Andorra
◯ Angola
◯ Anguilla
◯ Antarctica
◯ Antigua and Barbuda
◯ Argentina
◯ Armenia
◯ Aruba
◯ Australia
◯ Austria
◯ Azerbaijan
◯ Bahamas
◯ Bahrain
◯ Bangladesh
◯ Barbados
◯ Belarus
◯ Belgium
◯ Belize
◯ Benin
◯ Bermuda
◯ Bhutan
◯ Bolivia, Plurinational State of
◯ Bosnia and Herzegovina
◯ Botswana
◯ Bouvet Island
◯ Brazil
◯ British Indian Ocean Territory
◯ Brunei Darussalam
◯ Bulgaria
◯ Burkina Faso
◯ Burundi
◯ Cte dIvoire
◯ Cambodia
◯ Cameroon
◯ Canada
◯ Cape Verde
◯ Cayman Islands
◯ Central African Republic
◯ Chad
◯ Chile
◯ China
◯ Christmas Island
◯ Cocos (Keeling) Islands
◯ Colombia
◯ Comoros
◯ Congo
◯ Congo, the Democratic Republic of the
◯ Cook Islands
◯ Costa Rica
◯ Croatia
◯ Cuba
◯ Cyprus
◯ Czech Republic
◯ Denmark
◯ Djibouti
◯ Dominica
◯ Dominican Republic
◯ Ecuador
◯ Egypt
◯ El Salvador
◯ Equatorial Guinea
◯ Eritrea
◯ Estonia
◯ Ethiopia

◯ Falkland Islands (Malvinas)
◯ Faroe Islands
◯ Fiji
◯ Finland
◯ France
◯ French Guiana
◯ French Polynesia
◯ French Southern Territories
◯ Gabon
◯ Gambia
◯ Georgia
◯ Germany
◯ Ghana
◯ Gibraltar
◯ Greece
◯ Greenland
◯ Grenada
◯ Guadeloupe
◯ Guatemala
◯ Guernsey
◯ Guinea
◯ Guinea-Bissau
◯ Guyana
◯ Haiti
◯ Heard Island and McDonald Islands
◯ Holy See (Vatican City State)
◯ Honduras
◯ Hong Kong
◯ Hungary
◯ Iceland
◯ India
◯ Indonesia
◯ Iran, Islamic Republic of
◯ Iraq
◯ Ireland
◯ Isle of Man
◯ Israel
◯ Italy
◯ Jamaica
◯ Japan
◯ Jersey
◯ Jordan
◯ Kazakhstan
◯ Kenya
◯ Kiribati
◯ Korea, Democratic Peoples Republic of
◯ Korea, Republic of
◯ Kuwait
◯ Kyrgyzstan
◯ Lao Peoples Democratic Republic
◯ Latvia
◯ Lebanon
◯ Lesotho
◯ Liberia
◯ Libyan Arab Jamahiriya
◯ Liechtenstein
◯ Lithuania
◯ Luxembourg
◯ Macao
◯ Macedonia, the former Yugoslav Republic of
◯ Madagascar
◯ Malawi
◯ Malaysia
◯ Maldives
◯ Mali
◯ Malta
◯ Marshall Islands
◯ Martinique
◯ Mauritania
◯ Mauritius
◯ Mayotte

◯ Mexico
◯ Micronesia, Federated States of
◯ Moldova, Republic of
◯ Monaco
◯ Mongolia
◯ Montenegro
◯ Montserrat
◯ Morocco
◯ Mozambique
◯ Myanmar
◯ Namibia
◯ Nauru
◯ Nepal
◯ Netherlands
◯ Netherlands Antilles
◯ New Caledonia
◯ New Zealand
◯ Nicaragua
◯ Niger
◯ Nigeria
◯ Niue
◯ Norfolk Island
◯ Northern Mariana Islands
◯ Norway
◯ Oman
◯ Pakistan
◯ Palau
◯ Palestinian Territory, Occupied
◯ Panama
◯ Papua New Guinea
◯ Paraguay
◯ Peru
◯ Philippines
◯ Pitcairn
◯ Poland
◯ Portugal
◯ Qatar
◯ R union
◯ Romania
◯ Russian Federation
◯ Rwanda
◯ Saint Barthlemy
◯ Saint Helena, Ascension and Tristan da Cunha
◯ Saint Kitts and Nevis
◯ Saint Lucia
◯ Saint Martin (French part)
◯ Saint Pierre and Miquelon
◯ Saint Vincent and the Grenadines
◯ Samoa
◯ San Marino
◯ Sao Tome and Principe
◯ Saudi Arabia
◯ Senegal
◯ Serbia
◯ Seychelles
◯ Sierra Leone
◯ Singapore
◯ Slovakia
◯ Slovenia
◯ Solomon Islands
◯ Somalia
◯ South Africa
◯ South Georgia and the South Sandwich Islands
◯ Spain
◯ Sri Lanka
◯ Sudan
◯ Suriname
◯ Svalbard and Jan Mayen
◯ Swaziland
◯ Sweden
◯ Switzerland

◯ Syrian Arab Republic
◯ Taiwan, Province of China
◯ Tajikistan
◯ Tanzania, United Republic of
◯ Thailand
◯ Timor-Leste
◯ Togo
◯ Tokelau
◯ Tonga
◯ Trinidad and Tobago
◯ Tunisia
◯ Turkey
◯ Turkmenistan
◯ Turks and Caicos Islands
◯ Tuvalu
◯ Uganda
◯ Ukraine
◯ United Arab Emirates
◯ United Kingdom
◯ United States
◯ Uruguay
◯ Uzbekistan
◯ Vanuatu
◯ Venezuela, Bolivarian Republic of
◯ Vietnam
◯ Virgin Islands, British
◯ Wallis and Futuna
◯ Western Sahara
◯ Yemen
◯ Zambia
◯ Zimbabwe

State of residence	◯ ALABAMA ◯ ALASKA<br>◯ AMERICAN SAMOA ◯ ARIZONA<br>◯ ARKANSAS ◯ CALIFORNIA<br>◯ COLORADO ◯ CONNECTICUT<br>◯ DELAWARE ◯ DISTRICT OF COLUMBIA<br>◯ FLORIDA ◯ GEORGIA<br>◯ GUAM ◯ HAWAII ◯ IDAHO<br>◯ ILLINOIS ◯ INDIANA<br>◯ IOWA ◯ KANSAS ◯ KENTUCKY<br>◯ LOUISIANA ◯ MAINE<br>◯ MARYLAND ◯ MASSACHUSETTS<br>◯ MICHIGAN ◯ MINNESOTA<br>◯ MISSISSIPPI ◯ MISSOURI<br>◯ MONTANA ◯ NEBRASKA<br>◯ NEVADA ◯ NEW HAMPSHIRE<br>◯ NEW JERSEY ◯ NEW MEXICO<br>◯ NEW YORK ◯ NORTH CAROLINA<br>◯ NORTH DAKOTA ◯ OHIO<br>◯ OKLAHOMA ◯ OREGON<br>◯ PENNSYLVANIA ◯ PUERTO RICO<br>◯ RHODE ISLAND ◯ SOUTH CAROLINA<br>◯ SOUTH DAKOTA ◯ TENNESSEE<br>◯ TEXAS ◯ UTAH ◯ VERMONT<br>◯ VIRGIN ISLANDS ◯ VIRGINIA<br>◯ WASHINGTON ◯ WEST VIRGINIA<br>◯ WISCONSIN ◯ WYOMING
Gender	◯ Female<br>◯ Male<br>◯ Other
Race/Ethnicity (may check more than one)	☐ White<br>☐ Black or African American<br>☐ American Indian / Native Alaskan<br>☐ East Asian (incl. Chinese, Japanese, Korean)<br>☐ South / South-East Asian (incl. Bangladeshi, Indian, Pakistani, Sri Lankan)<br>☐ Native Hawaiian / Pacific Islander<br>☐ Arabic<br>☐ Other<br>☐ Unknown
Other race/ethnicity	______________________________
Hispanic ethnicity	◯ Hispanic/Latino<br>◯ Not Hispanic/Latino<br>◯ Unknown
Patient BMI category (in kg/m2)	◯ < 18.5 (Underweight)<br>◯ 18.5-24.9 (Normal weight)<br>◯ 25.0-29.9 (Pre-obesity)<br>◯ 30.0-34.9 (Obesity class I)<br>◯ 35.0-39.9 (Obesity class II)<br>◯ >39.9 (Obesity class III)<br>◯ Unknown

Liver transplantation questions

Has the patient had a liver transplantation?

◯ Yes
◯ No

What year was the liver transplantation performed?

◯ Unknown
◯ 2020
◯ 2019
◯ 2018
◯ 2017
◯ 2016
◯ 2015
◯ 2014
◯ 2013
◯ 2012
◯ 2011
◯ 2010
◯ 2009
◯ 2008
◯ 2007
◯ 2006
◯ 2005
◯ 2004
◯ 2003
◯ 2002
◯ 2001
◯ 2000
◯ 1999
◯ 1998
◯ 1997
◯ 1996
◯ 1995
◯ 1994
◯ 1993
◯ 1992
◯ 1991
◯ 1990
◯ 1989
◯ 1988
◯ 1987
◯ 1986
◯ 1985
◯ 1984
◯ 1983
◯ 1982
◯ 1981
◯ 1980
◯ 1979
◯ 1978
◯ 1977
◯ 1976
◯ 1975
◯ 1974
◯ 1973
◯ 1972
◯ 1971
◯ 1970
◯ 1969
◯ 1968
◯ 1967
◯ 1966
◯ 1965

Indication for liver transplant (select all that apply)

☐ Decompensated cirrhosis
☐ Hepatocellular carcinoma
☐ Acute liver failure
☐ Other

Other indication for liver transplantation	__________________________________
Underlying aetiology of liver disease (select all that apply)	☐ Non-alcoholic fatty liver disease (NAFLD)<br>☐ Alcohol-related liver disease (ALD)<br>☐ Hepatitis C virus (HCV)<br>☐ Hepatitis B virus (HBV)<br>☐ Autoimmune hepatitis (AIH)<br>☐ IgG4-related disease<br>☐ Primary biliary cholangitis (PBC)<br>☐ Primary sclerosing cholangitis (PSC)<br>☐ Hemochromatosis<br>☐ Wilson's disease<br>☐ Other
Other aetiology	__________________________________
Immunosuppression regimen at time of COVID 19 infection (select all that apply)	☐ Prednisone<br>☐ Tacrolimus<br>☐ Sirolimus<br>☐ Everolimus<br>☐ Cyclosporine<br>☐ Mycophenolate mofetil (MMF)<br>☐ Azathioprine<br>☐ Cyclophosphamide<br>☐ Other<br>☐ Unknown
What other immunosuppression medication was used at the time of COVID 19 infection?	__________________________________
Current prophylactic antimicrobial regimen (select all that apply)	☐ Trimethoprim/sulfamethoxazole or Co-trimoxazole<br>☐ Dapsone<br>☐ Pentamidine<br>☐ Acyclovir/valacyclovir<br>☐ Fluconazole<br>☐ Ganciclovir/valganciclovir<br>☐ Foscarnet<br>☐ Other<br>☐ Unknown<br>☐ None
Other prophylactic antimicrobial regimen	__________________________________

Does the patient have any of the following comorbidities (check all that apply)?

- ☐ Cardiovascular disease (coronary artery disease, heart failure, arrhythmia, etc.)
- ☐ Diabetes
- ☐ Asthma
- ☐ COPD
- ☐ Other Chronic Lung Disease (NOT asthma/COPD)
- ☐ Hypertension
- ☐ Non-HCC cancer
- ☐ History of stroke
- ☐ Chronic renal disease (CKD, etc.)
- ☐ Human immunodeficiency virus (HIV) infection
- ☐ Current cigarette smoker
- ☐ Current user of tobacco products other than cigarettes (vaping, etc)
- ☐ Current heavy alcohol use (>2 drinks/day for men, >1 drink/day for women)
- ☐ History of illicit drug use including injectable drugs or inhaled crack/cocaine but excluding marijuana

Chronic liver disease questions

Aetiology of liver disease (select all that apply)

- ☐ Non-alcoholic fatty liver disease (NAFLD)
- ☐ Alcohol-related liver disease (ALD)
- ☐ Hepatitis C virus (HCV)
- ☐ Hepatitis B virus (HBV)
- ☐ Autoimmune hepatitis (AIH)
- ☐ IgG4-related disease
- ☐ Primary biliary cholangitis (PBC)
- ☐ Primary sclerosing cholangitis (PSC)
- ☐ Hemochromatosis
- ☐ Wilson's disease
- ☐ Other

Other aetiology

____

Does the patient have cirrhosis?

- ◯ Yes
- ◯ No
- ◯ Unknown

Child Pugh grade prior to COVID 19 diagnosis?

- ◯ A
- ◯ B
- ◯ C
- ◯ Unknown

Did the patient have ascites prior to COVID 19 diagnosis?

- ◯ None
- ◯ Mild/moderate (diuretic responsive)
- ◯ Severe (diuretic refractory)
- ◯ Unknown

What was the worst grade of hepatic encephalopathy patient developed prior to COVID 19 diagnosis?	◯ None<br>◯ Grade 1 (trivial lack of awareness, shortened attention span)<br>◯ Grade 2 (lethargy, minimal disorientation, subtle personality change)<br>◯ Grade 3 (somnolence to semi-stupor but responsive to verbal stimuli, gross disorientation)<br>◯ Grade 4 (coma - unresponsive to verbal or noxious stimuli)<br>◯ Unknown
Has the patient ever had hepatocellular carcinoma?	◯ Yes<br>◯ No<br>◯ Unknown
What immunosuppression was the patient taking for IgG4-related disease at time of COVID 19 diagnosis (may check more than one)?	☐ None<br>☐ Corticosteroids<br>☐ Azathioprine<br>☐ Rituximab<br>☐ Other<br>☐ Unknown
Other immunosuppression for IgG4-related disease	__________________________________
Has the patient received steroids for alcoholic hepatitis recently (within 4 weeks of COVID 19 diagnosis)?	◯ Yes<br>◯ No<br>◯ Unknown
Treatment for primary biliary cholangitis (PBC) at time of COVID 19 diagnosis	☐ Ursodeoxycholic acid<br>☐ Obeticholic acid<br>☐ Fibrate<br>☐ Other<br>☐ No treatment<br>☐ Unknown
Other PBC treatments	__________________________________
Does the patient have inflammatory bowel disease (IBD)?	◯ Yes<br>◯ No<br>◯ Unknown
Immunosuppression medication(s) patient was taking for IBD at time of COVID 19 diagnosis	☐ Prednisone/prednisolone<br>☐ Budesonide<br>☐ Azathioprine<br>☐ Methotrexate<br>☐ Mycophenolate<br>☐ Infliximab<br>☐ Adalimumab<br>☐ Ustekinumab<br>☐ Vedolizumab<br>☐ Other<br>☐ None<br>☐ Unknown
Other immunosuppression for IBD	__________________________________

Treatment for autoimmune hepatitis (AIH) at time of COVID 19 diagnosis	☐ Prednisone/prednisolone<br>☐ Budesonide<br>☐ Azathioprine<br>☐ Mycophenolate<br>☐ Tacrolimus<br>☐ Other<br>☐ None<br>☐ Unknown
Other treatment for AIH	__________________________________
Hepatitis B surface antigen (HBsAg) positive	◯ Yes<br>◯ No<br>◯ Unknown
Treatment for HBV at the time of COVID 19 diagnosis	◯ Tenofovir<br>◯ Entecavir<br>◯ Interferon<br>◯ None<br>◯ Unknown
Did the patient have detectable hepatitis C virus (HCV) RNA at the time of or prior to COVID 19 diagnosis?	◯ Yes<br>◯ No<br>◯ Unknown
HCV genotype	◯ 1<br>◯ 2<br>◯ 3<br>◯ 4<br>◯ 5<br>◯ 6<br>◯ 7<br>◯ Unknown
Active treatment for HCV at the time of COVID 19 diagnosis	◯ Yes<br>◯ No<br>◯ Unknown
Does the patient have any of the following comorbidities (check all that apply)?	☐ Cardiovascular disease (coronary artery disease, heart failure, arrhythmia, etc.)<br>☐ Diabetes<br>☐ Asthma<br>☐ COPD<br>☐ Other Chronic Lung Disease (NOT asthma/COPD)<br>☐ Hypertension<br>☐ Non-HCC cancer<br>☐ History of stroke<br>☐ Chronic renal disease (CKD, etc.)<br>☐ Human immunodeficiency virus (HIV) infection<br>☐ Current cigarette smoker<br>☐ Current user of tobacco products other than cigarettes (vaping, etc)<br>☐ Current heavy alcohol use (>2 drinks/day for men, >1 drink/day for women)<br>☐ History of illicit drug use including injectable drugs or inhaled crack/cocaine but excluding marijuana

Laboratory data (leave fields blank if unknown)
• If COVID-19 suspected at presentation/admission then use recent pre-admission laboratory values as baseline.
• If hospital acquired COVID-19 suspected then please use first laboratory values obtained during hospitalisation as baseline.

Baseline serum sodium (mmol/L)	______________ (Before COVID 19 infection)
Nadir serum sodium (mmol/L)	______________ (During COVID 19 infection)
Creatinine units	○ µmol/L ○ mg/dl
Baseline serum creatinine	______________ (Before COVID 19 infection)
Peak serum creatinine	______________ (During COVID 19 infection)
Baseline prothrombin time (sec)	______________ (Before COVID 19 infection)
Peak prothrombin time (sec)	______________ (During COVID 19 infection)
Albumin units	○ g/dl ○ g/liter
Baseline albumin	______________ (Before COVID 19 infection)
Nadir albumin	______________ (During COVID 19 infection)
Total bilirubin units	○ µmol/L ○ mg/dl
Baseline total bilirubin	______________ (Before COVID 19 infection)
Peak total bilirubin	______________ (During COVID 19 infection)
Baseline alanine aminotransferase (ALT) (IU/L)	______________ (Before COVID 19 infection)

Peak alanine aminotransferase (ALT) (IU/L)

____
(During COVID 19 infection)

Baseline alkaline phosphatase (IU/L)

____
(Before COVID 19 infection)

Peak alkaline phosphatase (IU/L)

____
(During COVID 19 infection)

COVID 19 questions

Was this lab confirmed COVID 19 infection?

- ◯ Yes
- ◯ No
- ◯ Unknown

What symptoms did the patient have at the time of COVID 19 diagnosis?

- ◯ GI symptoms (abdominal pain, diarrhea, nausea, vomiting)
- ◯ Respiratory symptoms (shortness of breath, cough)
- ◯ Both GI and respiratory symptoms
- ◯ Neither GI or respiratory symptoms
- ◯ Unknown

Did patient test positive for influenza at time of COVID 19 infection?

- ◯ Yes
- ◯ No
- ◯ Unknown

What complications did the patient develop during COVID 19 infection?

- ☐ New or worsening ascites
- ☐ Spontaneous bacterial peritonitis
- ☐ Hepatic encephalopathy
- ☐ Non-variceal upper GI bleeding
- ☐ Variceal upper GI bleeding
- ☐ New requirement for renal replacement therapy (e.g. hemodialysis)
- ☐ Other

Other complications of COVID 19 infection

____

What was the grade of ascites during COVID 19 infection?

- ◯ Mild/moderate
- ◯ Severe
- ◯ Unknown

What was the worst grade of hepatic encephalopathy during COVID 19 infection (based on West Haven Criteria)?

- ◯ Grade 1 (trivial lack of awareness, shortened attention span)
- ◯ Grade 2 (lethargy, minimal disorientation, subtle personality change)
- ◯ Grade 3 (somnolence to semi-stupor but responsive to verbal stimuli, gross disorientation)
- ◯ Grade 4 (coma - unresponsive to verbal or noxious stimuli)
- ◯ Unknown

Did the patient receive specific antiviral treatment for COVID 19 infection?

- ◯ Yes
- ◯ No
- ◯ Unknown

Which of the following treatment(s) did patient receive for COVID 19 (select all that apply)?	☐ Remdesivir<br>☐ Tocilizumab<br>☐ Lopinovir/ritonavir<br>☐ Chloroquine/hydroxychloroquine<br>☐ Ribavirin<br>☐ Interferon-alpha<br>☐ Other<br>☐ Unknown
Other type of antiviral treatment(s) received for COVID-19	__________________________________
What was the reason the patient did not receive COVID 19 antiviral treatment (select all that apply)?	☐ Elevated liver enzymes<br>☐ Underlying liver fibrosis/cirrhosis<br>☐ Other contraindication (eg AKI)<br>☐ Treatment not available<br>☐ Other<br>☐ Unknown
What was the reason the patient did not receive COVID 19 antiviral treatment?	__________________________________
Did the patient die?	◯ Yes<br>◯ No<br>◯ Unknown
What was the primary cause of death?	◯ Liver-related complications<br>◯ COVID 19-related lung disease<br>◯ Cardiogenic shock<br>◯ Other
Other cause of death	__________________________________
Has the patient been hospitalized?	◯ Yes<br>◯ No<br>◯ Unknown
Has the patient been discharged from the hospital?	◯ Yes<br>◯ No<br>◯ Unknown
Length of hospital stay (days)	__________________________________
Did patient receive invasive ventilation?	◯ Yes<br>◯ No<br>◯ Unknown
Did the patient receive non-invasive ventilation?	◯ Yes<br>◯ No<br>◯ Unknown
Was the patient admitted to an intensive care unit?	◯ Yes<br>◯ No<br>◯ Unknown

Why was the patient not admitted to an intensive care unit?	◯ Disease not severe enough<br>◯ Disease was severe enough but limited availability of intensive care unit<br>◯ Disease was severe enough but escalation to intensive care unit not felt to be appropriate.<br>◯ Unknown
